# A Targeted, Bioinert LC–MS/MS Method for Sensitive, Comprehensive Analysis of Signaling Lipids

**DOI:** 10.1021/acs.analchem.4c01388

**Published:** 2024-05-25

**Authors:** Stefanie Rubenzucker, Mailin-Christin Manke, Rainer Lehmann, Alice Assinger, Oliver Borst, Robert Ahrends

**Affiliations:** † Department of Analytical Chemistry, University of Vienna, 1090 Vienna, Austria; ‡ Vienna Doctoral School in Chemistry, University of Vienna, 1090 Vienna, Austria; § DFG Heisenberg Group Cardiovascular Thromboinflammation and Translational Thrombocardiology, University of Tübingen, 72076 Tübingen, Germany; ∥ Department of Cardiology and Angiology, University of Tübingen, 72076 Tübingen, Germany; ⊥ Institute for Clinical Chemistry and Pathobiochemistry, Department for Diagnostic Laboratory Medicine, University Hospital Tübingen, 72076 Tübingen, Germany; # Department of Vascular Biology and Thrombosis Research, Centre of Physiology and Pharmacology, Medical University of Vienna, 1090 Vienna, Austria

## Abstract

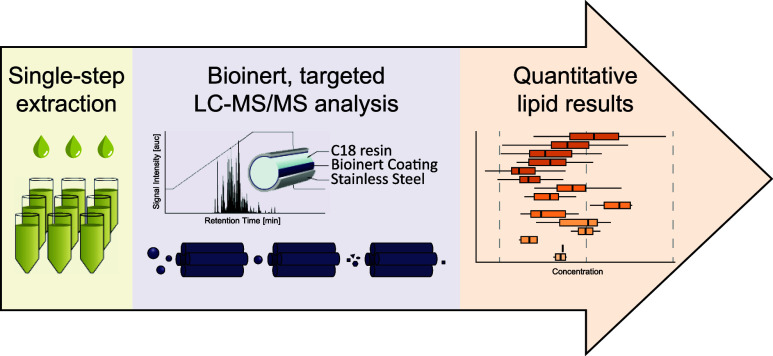

Signaling lipids are key players in cellular processes. Despite their importance, no method currently allows their comprehensive monitoring in one analytical run. Challenges include a wide dynamic range, isomeric and isobaric species, and unwanted interaction along the separation path. Herein, we present a sensitive and robust targeted liquid chromatography-mass spectrometry (LC–MS/MS) approach to overcome these challenges, covering a broad panel of 17 different signaling lipid classes. It involves a simple one-phase sample extraction and lipid analysis using bioinert reversed-phase liquid chromatography coupled to targeted mass spectrometry. The workflow shows excellent sensitivity and repeatability in different biological matrices, enabling the sensitive and robust monitoring of 388 lipids in a single run of only 20 min. To benchmark our workflow, we characterized the human plasma signaling lipidome, quantifying 307 endogenous molecular lipid species. Furthermore, we investigated the signaling lipidome during platelet activation, identifying numerous regulations along important lipid signaling pathways. This highlights the potential of the presented method to investigate signaling lipids in complex biological systems, enabling unprecedentedly comprehensive analysis and direct insight into signaling pathways.

## Introduction

**Figure 1:**
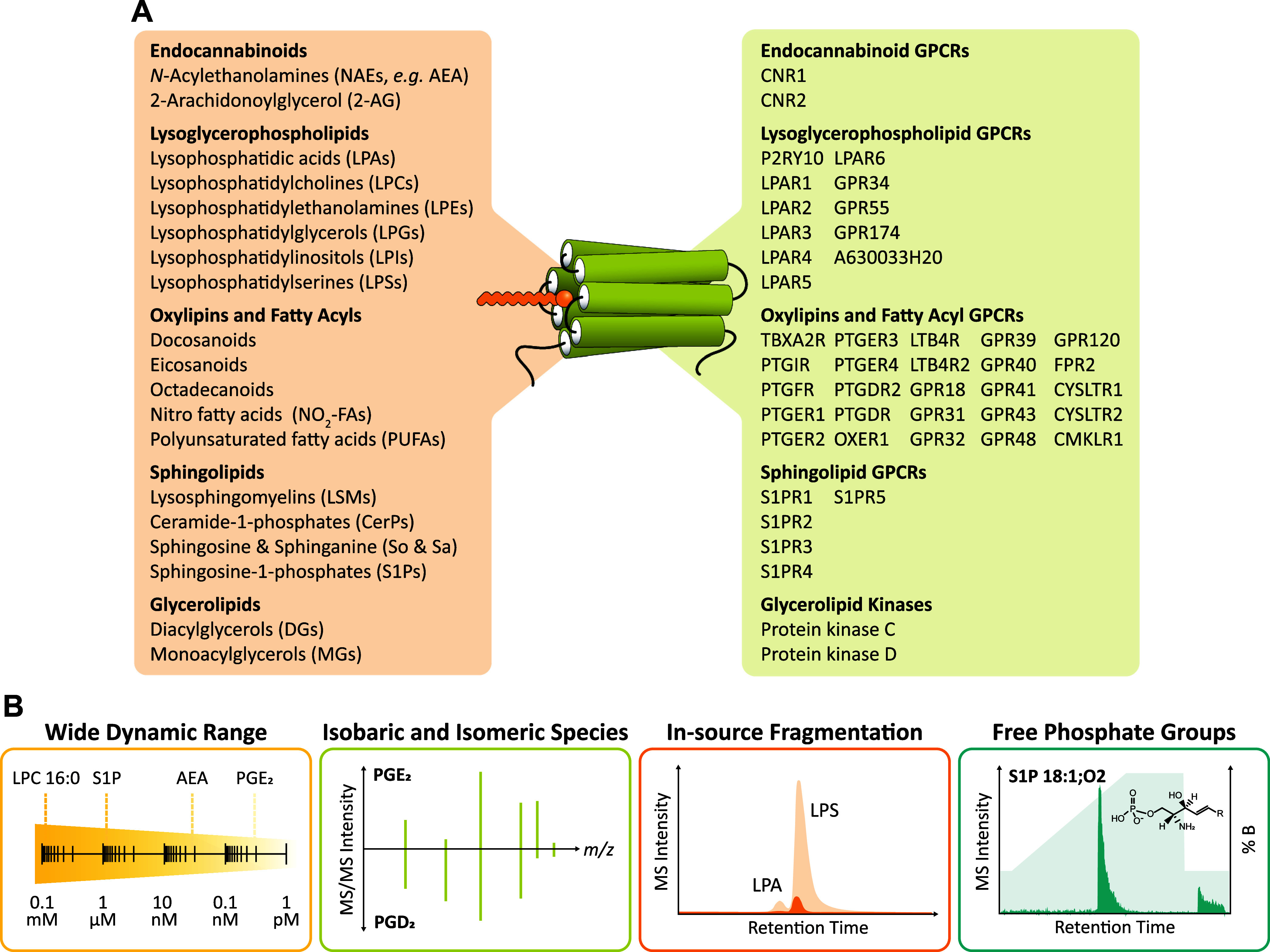
(A) Overview of different groups of signaling lipids and the GPCRs and kinases they act on. (B) Challenges associated with the comprehensive analysis of signaling lipids.

Signaling lipids are a diverse group of molecules that have recently garnered significant attention due to their essential roles in various physiological and pathological processes.^1−7^ These lipids exhibit remarkable chemical diversity and are synthesized in response to different stimuli, with glycero(phospho)lipids and sphingolipids serving as the precursors for most signaling lipids,^8−10^ including oxylipins, lysoglycerophospholipids, and endocannabinoids. In contrast to their precursors which mainly serve as structural and energy-building blocks in cells,^11^ many lipids with signaling capacity can directly modulate intracellular signaling pathways and often exert their regulatory function by directly binding to specific receptors like G protein-coupled receptors (GPCRs)^2,4,5^ or acting on other regulatory proteins like kinases^1,12^ (Figure 1A).

Based on their structure, signaling lipids can be classified into different groups. Oxylipins are a superfamily of potent lipid mediators derived from polyunsaturated fatty acids (PUFA) through enzymatic and nonenzymatic oxygenation reactions.^8,13^ They play critical roles in inflammation, endothelial function, and thrombosis and can indicate oxidative stress.^8,9,14,15^ Like PUFAs, lysoglycerophospholipids are generated through the hydrolysis of glycerophospholipids and are another group of lipids involved in inflammation, immune function, and cell proliferation.^3,16,17^ Representing one of the largest groups of signaling lipids,^18^ they can be further categorized into six subgroups based on their corresponding glycerophospholipid precursors: lysophosphatidic acids (LPA) and cyclic LPAs (CPA), lysophosphatidylcholines (LPC), lysophosphatidylethanolamines (LPE), lysophosphatidylglycerols (LPG), lysophosphatidylinositols (LPI), and lysophosphatidylserines (LPS). LPAs, the simplest lysoglycerophospholipid species, have been extensively studied and have been shown to influence physiological processes by acting on six dedicated GPCRs (LPAR1-6).^4^ Similarly, endocannabinoids such as anandamide (AEA) and 2-arachidonoylglycerol (2-AG) serve as potent immune and neuromodulators by acting on the GPCRs CB1 and CB2 of the endocannabinoid system^2,10,19^ and sphingosine-1-phosphates (S1P) mediate cell survival, migration, and immune response by acting on five dedicated GPCRs (S1PR1-5).^5,20^ S1Ps are part of a larger group of sphingoid-based lipid messengers, including the sphingoid bases sphingosine (So) and sphinganine (Sa), lysosphingomyelins (LSM), and ceramide-1-phosphates (CerP). While all lipids mentioned thus far mainly exert their effects by acting on GPCRs, certain lipids mediate biological processes by acting on kinases. This is the case for diacylglycerols (DGs), which have been reported to play an important role in hemostasis^12^ and metabolic processes like insulin resistance^21^ by activating protein kinase C.

Comprehensive and accurate methods for identifying and quantifying signaling lipids are essential to understand their role in biological processes. Over the past years, liquid chromatography–mass spectrometry (LC–MS) based methods have emerged as the gold standard for signaling lipid analysis.^8,15^ However, analyzing signaling lipids presents various challenges including a wide concentration range,^8,22,23^ isobaric and isomeric lipid species,^8^ in-source fragmentation,^24^ and chromatographic difficulties with lipids containing free phosphate groups like S1Ps, CerPs, and LPAs^25^ (Figure 1B). Currently, dedicated methods are employed for analyzing different signaling lipid classes. Reversed-phase (RP)-LC is the method of choice for separating oxylipins.^8,26^ However, lipids with free phosphate groups exhibit extensive peak tailing and carryover issues in RP-LC.^25^ Metal chelators like ethylenediaminetetraacetate (EDTA), pseudo ion-pairing reagents like orthophosphoric acid (PA), or hydrophilic interaction liquid chromatography (HILIC) are employed to address these challenges.^25,27−29^ However, HILIC fails to resolve isomeric oxylipins, and additives like EDTA or PA lead to significant ion suppression in negative ionization mode.^29^ Thus, there is a strong need for a comprehensive LC–MS method capable of simultaneously detecting and quantifying a large number of relevant signaling lipid classes in a single LC–MS run.

We herein report the development of a targeted liquid chromatography-tandem mass spectrometry (LC–MS/MS) method covering 388 signaling lipids from 17 lipid classes. Detection of lipids with free phosphate groups is highly improved and the carryover minimized using bioinert column hardware, an optimized solvent system, and a biocompatible LC system. Furthermore, sub-2 μm C18 resin enables excellent isomer separation of oxylipin isomers like PGD_2_/PGE_2_. The workflow was validated in plasma and platelet matrices, achieving high repeatability and lower limits of quantification (LLOQs) in the low nanomolar range. Finally, we used the established method to study the plasma signaling lipidome and the changes of signaling lipids during platelet activation, highlighting the broad applicability of our method.

## Experimental Section

### Materials

Acetonitrile (ACN), methanol (MeOH), and water were purchased in LC–MS grade from Biosolve (Valkenswaard, The Netherlands). Ammonium acetate (NH_4_Ac), calcium chloride (CaCl_2_), chloroform (CHCl_3_), citric acid, glucose, 2-[4-(2-hydroxyethyl)piperazin-1-yl]ethanesulfonic acid (HEPES), phosphoric acid (PA), potassium chloride (KCl), sodium hydrogen carbonate (NaHCO_3_), sodium chloride (NaCl), sodium dihydrogen phosphate (NaH_2_PO_4_), and *tert*-butyl methyl ether (MTBE) were obtained from Sigma-Aldrich (Steinheim, Germany) and acetic acid (HAc) and ethyl acetate (EtOAc) from Carl Roth (Karlsruhe, Germany). 1-Butanol (BuOH) and isopropanol (IPA) were purchased from Merck (Darmstadt, Germany) and ethanol (EtOH), and potassium hydrogen phosphate from Supelco (Bellefonte, USA). Collagen-related peptide (CRP) was obtained from Richard Farndale (University of Cambridge, UK) and thrombin from human plasma was purchased from Roche (Mannheim, Germany). All lipid standards were purchased from Cayman Chemical (Ann Arbor, USA) and Avanti Polar Lipids (Alabaster, USA). A detailed list is provided in the Supporting Information (Table S1).

### Platelet and Plasma Sample Generation

Platelets were obtained from 10- to 12-week-old male C57BL/6 mice, and plasma was obtained from 19 healthy volunteers (63% female, 37% male; mean age: 38 ± 12 years, no medication for ≥2 weeks). The detailed sample generation can be found in Text S1 in the Supporting Information.

### Evaluation of Extraction Protocols

Three different extraction protocols adapted from literature were tested: BuEt two-phase extraction,^15^ BuMe one-phase extraction,^30,31^ and MMC one-phase extraction.^32^ The detailed extraction protocols can be found in Text S2 in the Supporting Information. To ensure better comparability between the protocols, they were scaled to use the same amount of organic solvent in the first extraction step (1 mL). All sample processing steps were carried out on ice and using ice-cold solvents. Dried lipid extracts were reconstituted in 50 μL of H_2_O/IPA/BuOH 69/23/8 (v/v/v) + 25 nM CUDA (used as the system standard), centrifuged at 8 °C and 21,000*g* for 5 min and analyzed using LC–MS/MS. Extractions were carried out in triplicate. The extraction recovery and matrix effect were determined by spiking an internal standard (IS) mix (Table S2) pre- or postextraction or into the neat solvent and calculated as follows
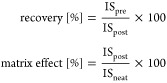


### Extraction of Plasma and Platelet Samples

The MMC one-phase extraction protocol was used to extract human plasma and murine platelets and their releasate. For human plasma, 100 μL aliquots were extracted. For murine platelets, one sample corresponds to 10^8^ platelets. The IS mixtures used for the respective samples can be found in Table S3.

### Method Optimization

For scheduled single reaction monitoring (sSRM) assay development, a QTrap 6500+ mass spectrometer (AB Sciex, Darmstadt, Germany) coupled to a Vanquish Flex UHPLC system (Thermo Scientific, Bremen, Germany) was operated in polarity switching mode. Declustering potential, collision energy (CE), and collision cell exit potential (CXP) were optimized via direct infusion of at least one standard per lipid class. The most abundant and, if possible, unique SRM transition was chosen for each analyte.

For chromatographic optimization, a mix of 17 different standards was used (Table S4). Three C18 columns (150 × 2.1 mm, 1.9 μm particle size), only differing in the material of the column body, were tested: YMC-Triart, YMC-Triart metal-free, and YMC-Accura Triart (YMC Europe, Dinslaken, Germany). The tested eluent systems and gradients can be found in Table S5.

Electrospray ion source parameters were optimized for the final LC method using the standard mixture previously described. Optimized values included voltage, source temperature, gas 1, gas 2, curtain gas, and collision gas.

### LC–MS/MS Method for Signaling Lipid Analysis

The final sSRM method consists of a 20 min run on the YMC-Accura Triart C18 column (150 × 2.1 mm, 1.9 μm particle size, YMC Europe, Dinslaken, Germany) fitted with a Vanquish MP35N passive preheater. The column compartment and autosampler are kept at 45 and 8 °C, respectively. Gradient elution is carried out at 0.4 mL/min with eluent A consisting of H_2_O/ACN 80/20 (v/v) and eluent B consisting of IPA/ACN/H_2_O 60/35/5 (v/v), both containing 0.5 mM NH_4_Ac and 0.2% HAc. The linear gradient is as follows: 0–1 min 30% B, 1–11 min increase to 100% B, hold at 100% B for 11–16 min, return to 30% B at 16.1 min, and hold for 3.9 min for re-equilibration. The injection volume is 5 μL, and the injector needle is automatically washed with IPA/ACN 9/1 (v/v) + 0.2% HAc + 5 μM PA before and after each injection. The optimized MS source parameters for the finalized LC method can be found in Table S6. The final sSRM method covers 388 lipid species. Monitored transitions, their corresponding retention time, and the respective internal standard used for quantification can be found in Table S7. To ensure at least 8–10 data points per peak for accurate quantification, the cycle time was set to 1 s, and the settling time was set to 20 ms. At the same time, the sSRM detection window was individually specified for each lipid species, and the minimum dwell time was set to 10 ms.

### Method Characterization

The method was characterized in neat solvent and three different sample matrices (human plasma and murine platelets and their releasates) regarding sensitivity, linearity, precision, matrix effect, carryover, and repeatability using an IS mix described in Table S8. Details on all evaluated parameters can be found in Text S3.

### Data Analysis

Details on the employed lipid nomenclature can be found in Table S9. Peak areas were integrated using Skyline 22.2.0.315,^33^ and quantification and statistical analysis were performed using R 4.3.2^34^/R Studio 2023.9.1.494^35^ and KNIME.^36^ Lipid spaces were calculated using LipidSpace.^37^

## Results and Discussion

Signaling lipids are a diverse and complex group of analytes with distinct physicochemical properties. Although individual lipid classes can be analyzed using tailored LC–MS methods,^15,25−28^ achieving comprehensive analysis of signaling lipids in one single LC–MS method is challenging due to the distinct analytical properties of each lipid class. The main challenges include reducing the interaction of free phosphate groups of lipids like CerPs with metal surfaces, ensuring a stable charge state of zwitterionic molecules such as S1Ps, and separating isobaric and isomeric species of, *e.g*., oxylipins from each other (Figure 1B). By thoroughly optimizing mass spectrometric and chromatographic parameters, we addressed these challenges and developed a robust, comprehensive LC–MS/MS method that quantifies 388 physicochemically very diverse signaling lipids in one analytical run.

### Chromatographic Optimization

A mix of 17 standards was utilized to establish a robust LC–MS/MS method. Initial tests were conducted on a C18 column, YMC-Triart, using a 20 min run employing a linear gradient with 0.1% acetic acid (HAc) as an eluent additive. Most lipids, including oxylipins, PUFAs, and endocannabinoids, showed good separation and signal intensity, whereas analytes with free phosphate groups, such as S1P, CerP, and LPA, exhibited strong peak tailing and pronounced carryover. Especially LPA and CerP hardly eluted throughout the 20 min run due to the coordination of the analytes’ free phosphate groups to the metal ions of the stainless-steel column body.^25^ Some studies have utilized chelating or ion-paring compounds such as EDTA and PA as eluent additives to overcome these issues.^25,29^ However, these additives pose challenges for MS analysis in negative ionization mode as they are nonvolatile and highly ionizable, leading to significant ion suppression effects.

**Figure 2:**
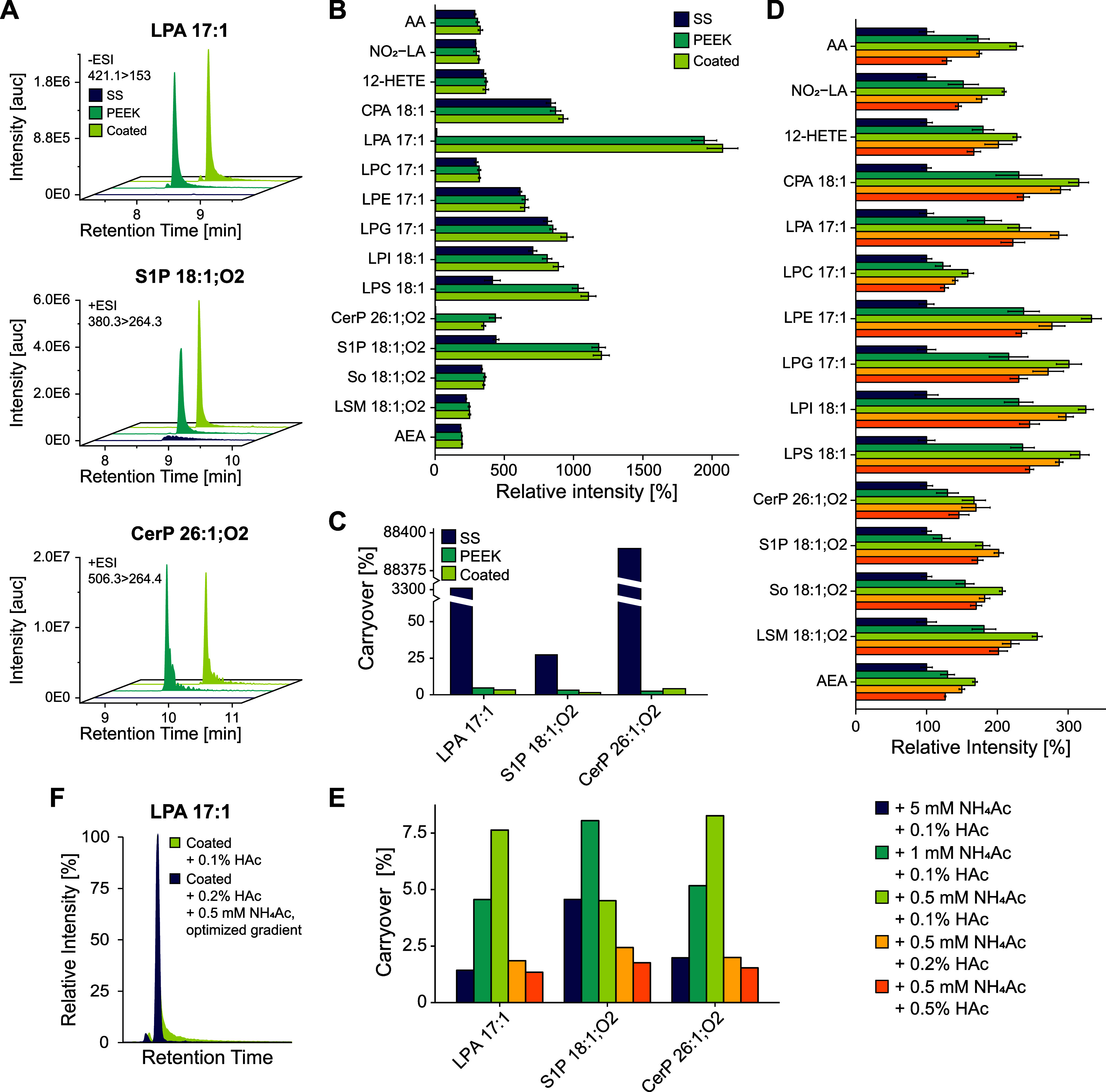
Development of a comprehensive LC method for signaling lipid analysis. (A) Extracted ion chromatograms (XICs) of free phosphate group-containing analytes LPA, S1P, and CerP. Three different columns only differing in the type of column hardware were tested using 0.1% HAc as an eluent additive. “SS” (stainless steel) corresponds to YMC-Triart, “PEEK” to YMC-Triart metal-free, and “coated” to YMC-Accura Triart. (B) Relative signal intensities for the three tested columns (*n* = 4). (C) Carryover of free phosphate group-containing analytes in dependency of the column coating. Carryover was calculated as the ratio between the peak area of the blank injection after the standard mix injection and the peak area of the standard mix injection itself and is expressed as a percentage. (D) Signal intensities for different salt and acid concentrations using the YMC-Accura Triart column (*n* = 4). (E) Carryover of LPA, S1P, and CerP using the YMC-Accura Triart column and different salt and acid concentrations. (F) Example of improved peak shape of LPA 17:1 after final method optimization.

To overcome the problem of metal coordination without compromising ionization efficiency, two different bioinert columns with the same column material and dimensions as the initially tested YMC-Triart but different types of column hardware were tested. While the YMC-Triart has a conventional stainless-steel body, the YMC-Triart metal-free and YMC-Accura Triart are characterized by a bioinert column surface. The YMC-Triart metal-free has a PEEK-lined stainless-steel column body, whereas the column hardware of the YMC-Accura Triart consists of stainless-steel column body with a bioinert coating. Indeed, both bioinert columns showed significant improvement for LPA, S1P, and CerP, yielding higher signal intensities, better peak shapes, and a carryover below 10% (Figure 2A–C). As expected, signal intensities for analytes containing no (free) phosphate groups were similar for all three tested columns. However, it is essential to note that the bioinert coating/lining type influences the chromatographic resolution. The PEEK-lined column showed a slightly lower resolution for all analyzed lipid species, which is especially disadvantageous when separating isomeric species without unique MS/MS fragments like PGD_2_/PGE_2_ (Figure S1). Ultimately, the YMC-Accura Triart was selected for further method development as it provided better resolution and slightly outperformed the PEEK-lined YMC-Triart metal-free column for most analytes regarding signal intensity.

Different salt and acid concentrations were tested to further reduce the carryover of free phosphate group-containing analytes (Figure 2D,E). In lipidomics, counterions such as NH_4_ salts are often used as pseudo ion-pairing agents to reduce the nonspecific adsorption and binding effects of negatively charged analytes.^38^ Furthermore, high acid concentrations have also been shown to decrease the carryover of challenging analytes like S1P.^39^ However, high salt and acid concentrations can decrease the ionization efficiency of other lipid groups like oxylipins (Figure 2D). The best compromise between reduced carryover and only minor loss of signal intensity was achieved using eluents containing 0.5 mM NH_4_Ac and 0.2% HAc. Lastly, the eluent composition and gradient starting conditions were adapted to enable efficient separation (Figure 2F) while also ensuring adequate washing of the column to prevent the accumulation of very apolar lipids like triacylglycerolipids (TGs). Additionally, the column oven temperature was reduced to 45 °C to prolong the column lifespan while still operating within the maximum pressure limit of the column. The final method ensures excellent separation and repeatability within its 20 min runtime. This is especially important for oxylipin isomers without unique MS/MS fragments and lysoglycerophospholipids with different headgroups but the same fatty acyl moiety (Figure S2), as in-source fragmentation of, *e.g*., LPS 18:1 can lead to LPA 18:1 artifact peaks.

### Building a Comprehensive Signaling Lipid LC–MS/MS-Method

Targeted mass spectrometry enables the sensitive and robust quantification of signaling lipids.^8,15,22,38^ However, to achieve a high coverage of lipid species the method has to feature very narrow acquisition windows for each monitored transition, making diligent optimization of the final lipid target list crucial. To obtain a comprehensive target list, an in-depth screening procedure employing the previously described optimized LC method was applied to two different sample types with unique lipid profiles (human plasma and murine platelets). Lipids exhibiting distinct fragmentation patterns (lysoglycerophospholipids, sphingolipids, glycerolipids, and endocannabinoids) were identified by monitoring characteristic MS/MS fragments in SRM mode in negative and/or positive ionization mode(s). The monitored SRM transitions were created using LipidCreator^40^ or were taken from reference spectra from LIPIDMAPS.^41^ For confident lipid identification at least two fragments had to be identified per lipid species, and the elution order within a lipid subclass had to fit the ECN model^42^ (Figure S3). The lysoglycerophospholipid target list was further extended by exploiting the correlation between the chromatographic retention time and the lipid headgroup and fatty acyl motive,^43^ thereby allowing the retention time prediction of lipid species not identified in the initial screening procedure. Lipid species that do not exhibit building block-like fragmentation patterns (oxylipins, nitrosylated fatty acids, and PUFAs) were identified by matching the retention time and MS/MS fragments to those of commercially available standards. The final LC–MS/MS method covers 388 lipids from 17 lipid classes. The monitored transitions and their corresponding retention time can be found in Table S7.

### Evaluation of Different Lipid Extraction Protocols

**Figure 3:**
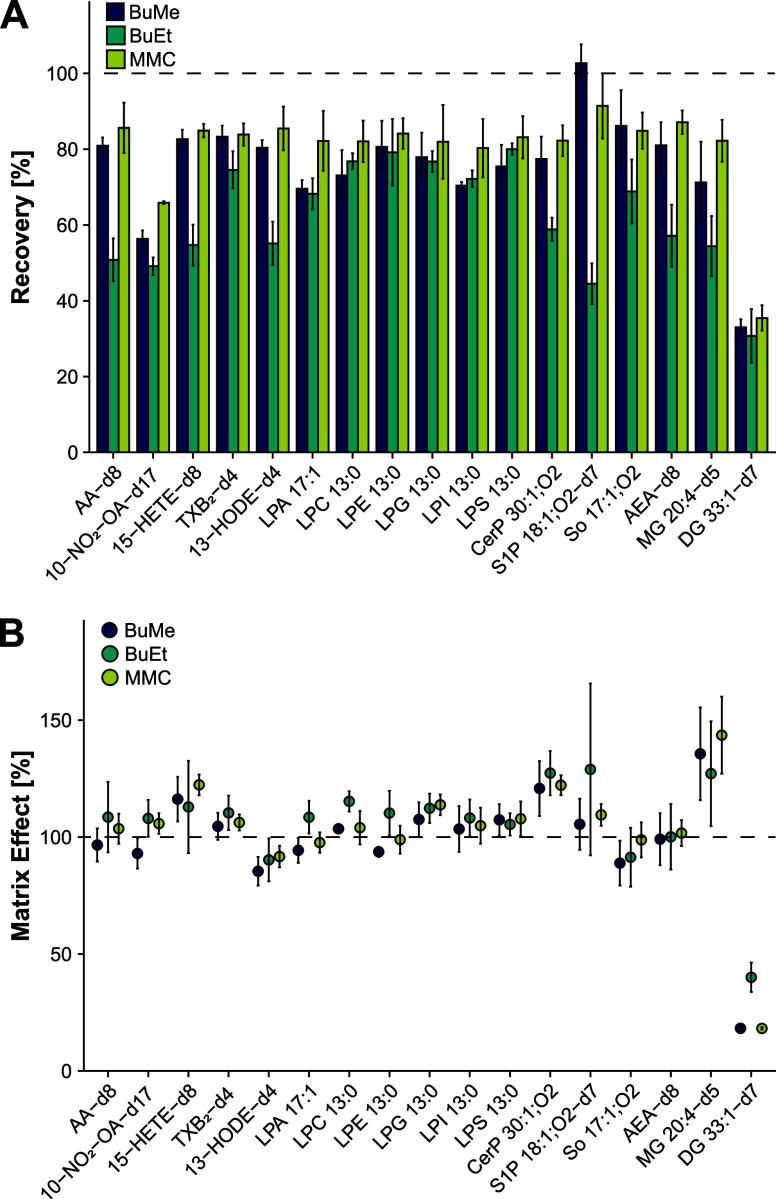
Evaluation of different extraction protocols. Two monophasic (BuMe^30,31^ and MMC^32^) and one biphasic (BuEt^15^) extraction methods were tested to evaluate their suitability for signaling lipid analysis (*n* = 3). (A) Recovery and (B) matrix effect were determined by spiking plasma samples with an internal standard mixture pre- and postextraction, where 100% corresponds to complete analyte recovery and no matrix effect.

Simultaneous analysis of nonpolar lipids such as DGs, very polar molecules such as LPAs, and zwitterionic lipids such as S1Ps requires the selection of a suitable extraction procedure to prevent the loss of low-abundant signaling lipids. To identify a suitable protocol for the extraction of all lipid classes of interest, three previously reported extraction methods were adapted and tested in plasma: BuMe (butanol/methanol, monophasic),^30,31^ BuEt (acidified butanol/ethyl acetate, biphasic),^15^ and MMC (methanol/MTBE/chloroform, monophasic).^32^ Although all three protocols performed similarly in terms of recovery for some lipid groups (*e.g*., lysoglycerophospholipids and DGs), the two monophasic extraction methods clearly outperformed the BuEt protocol for oxylipins, PUFAs, CerPs, S1Ps, and endocannabinoids (Figure 3A). The average extraction recoveries over all analytes were 80, 75, and 62%, and ranged from 35 to 91, 33–103, and 31–80%, for MMC, BuMe, and BuEt, respectively. Furthermore, 88% of the analytes had an extraction recovery of ≥80% for the MMC protocol, whereas for BuMe, this threshold was only reached for 47% of the analytes. For the BuEt protocol, the ≥80%-threshold was reached for only one analyte (6%), further corroborating its inferior performance compared to the monophasic extraction protocols. Although the three tested extraction protocols performed quite differently regarding extraction recovery, the matrix effect across the different extraction methods was similar for almost all examined analytes (Figure 3B). Most analytes were not significantly influenced by the sample matrix, with 76, 76, and 71% of the analyzed lipids showing matrix effects ≤20% for MMC, BuMe, and BuEt, respectively. The most notable exception are DGs, which show the most pronounced matrix effect while exhibiting the worst extractability. It is also the only lipid class where the biphasic BuEt extraction significantly outperforms the two one-phase extraction protocols in terms of matrix effect. Most likely, the BuEt protocol is more effectively depleting very apolar lipid species, thereby reducing matrix interferences in the last part of the chromatographic gradient where DGs are eluting. Ultimately, MMC was chosen as the most suitable extraction method with respect to all covered lipid classes due to its high recoveries and ease of use, guaranteeing adequate lipid extraction from the sample.

### Method Characterization

**Figure 4:**
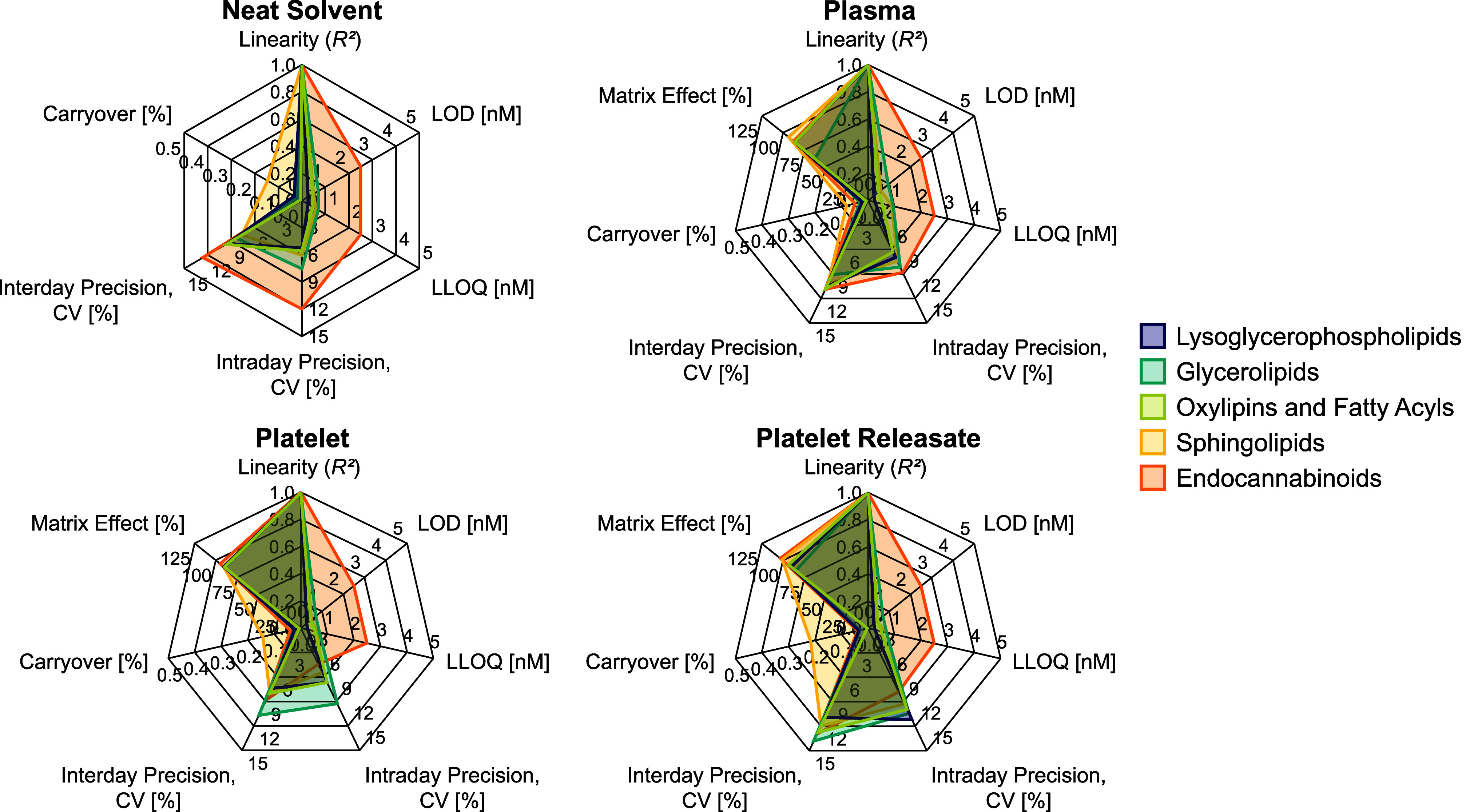
Method characteristics evaluated in neat solvent, plasma, platelet, and platelet releasate (*n* = 3). Values are given as an average for each lipid category. Detailed results can be found in Tables S10–S12. CV = coefficient of variation, LOD = limit of detection, LLOQ = lower limit of quantification.

To ensure that the established method is fit for purpose, its performance was characterized in neat solvent and three different biological matrices (plasma, platelet, and platelet releasate). The method characteristics for all matrices can be found in Tables S10–S13, and average values for each lipid category are represented in radar charts in Figure 4.

Covering a wide concentration range is essential for reliable signaling lipid analyses as concentrations range from subnanomolar for, *e.g*., oxylipins^9,26^ and LPAs^27^ to high micromolar concentrations for more abundant species like PUFAs and DGs.^9,22,38^ Furthermore, low LLOQs are needed to quantify low-abundant lipids. Overall, the method showed excellent linearity over 3–4 orders of magnitude, with 25 of the 27 investigated lipids having *R*^2^ > 0.99 in all tested matrices (Figures S4–S7). The only two lipids with an *R*^*2*^ slightly below 0.99 were sphingosine and sphinganine in plasma. All investigated lipids showed LLOQs in the low nanomolar range, with 22 analytes having LLOQs in the subnanomolar range in all matrices. These values are in a similar range as previously reported for methods only covering a small subset of signaling lipids (*e.g.*, only oxylipins, sphingolipids, or LPA).^8,15,25,27^ This highlights the potential of the herein reported method for the quantification of an unprecedentedly extensive set of signaling lipids while at the same time ensuring the necessary sensitivity to detect even low-abundant lipid species.

The developed method also showed good repeatability, with 100% of the investigated lipids having a relative standard deviation ≤15% for inter- and intraday precision in all investigated matrices. Since sSRM methods entail the predefinition of fixed acquisition windows for each analyte, retention time stability is another crucial characteristic of the method. The retention time variability was assessed over multiple batches including neat standard injections as well as plasma and platelet samples measured over several weeks (Table S14 and Figure S8). Overall, the observed retention time shifts were well below 1% for all analytes, demonstrating excellent repeatability of the chromatographic method. The lowest retention time shift was observed for DG 15:0–18:1-d7 with only 0.08% or 0.6 s, whereas AEA-d8 had the highest variability with 0.71% or 4.2 s.

As mentioned in the section *Chromatographic Optimization*, ensuring minimal carryover, especially for free phosphate-containing lipids, is highly important. To demonstrate that the established method is suitable for analyzing biological samples, carryover was assessed by injecting a blank sample after the highest calibrator. Except for the two free phosphate-containing analytes LPA 17:1 and CerP 18:1;O2/12:0, which showed a slightly higher but still acceptable carryover of 0.14 and 0.28%, respectively, no lipids showed noticeable carryover.

Lastly, we assessed the matrix effect for plasma and platelet samples. Most analytes showed low matrix effects across all sample types. The most noteworthy exception is DG 15:0–18:1-d7 in plasma, with a matrix effect of 17.48% (with 100% corresponding to no matrix effect). Since DGs elute at the end of the chromatographic gradient, the pronounced matrix effect is most likely caused by coeluting apolar species, *e.g*., TGs and cholesteryl esters (CEs), coextracted during the extraction process. However, since adequate surrogate standards are used for quantification, the bias introduced through different matrices is compensated for.

### Investigating the Plasma and Platelet Signaling Lipidome

To demonstrate the wide applicability of our method, we applied our LC–MS/MS workflow to two sample types with very different inherent challenges: plasma, having a very “crowded” lipidome with highly concentrated apolar species, and resting and stimulated platelets, exhibiting a highly dynamic lipidome.

**Figure 5:**
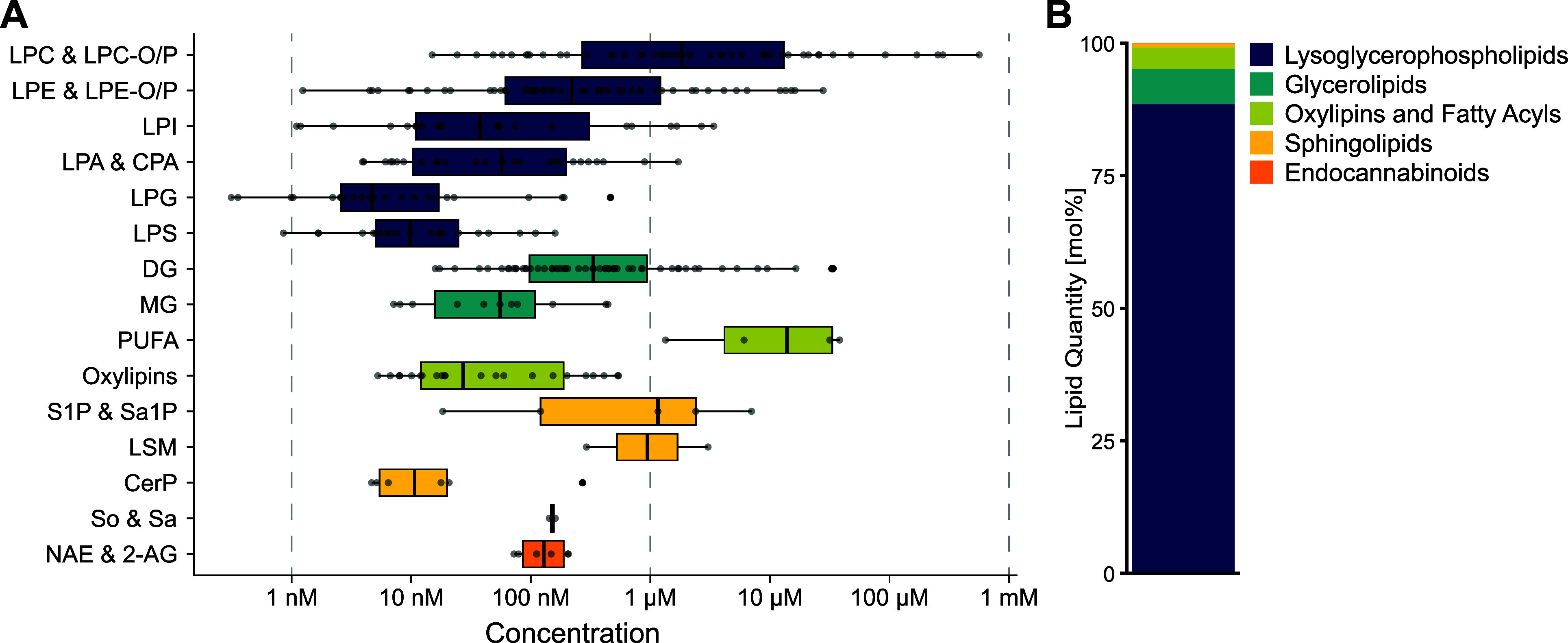
Composition of the human plasma signaling lipidome (*n* = 19). (A) 307 lipid species from 5 different lipid categories spanning a dynamic range of 6 orders of magnitude were quantified. Each boxplot is composed of all quantified lipid species within the respective lipid group, where each dot corresponds to a molecular lipid species. A detailed list of all quantified molecular lipid species and their respective concentrations can be found in the Supporting Information. (B) Relative lipid distribution of signaling lipids in plasma.

To benchmark our method, plasma of 19 healthy human subjects was analyzed employing our LC–MS/MS approach. Lipid concentrations were estimated using single-point calibration with deuterated or odd-chain internal standards representative of the different lipid classes (Table S3). A total of 307 lipids were quantified on the molecular species level (Table S15), with concentrations spanning 6 orders of magnitude (Figure 5A). Lysoglycerophospholipids showed the broadest concentration range, with levels ranging from very low nM (LPG) to high μM (LPC). DG, PUFA, LPC, and LPE lipid species showed the highest concentrations, whereas other glycerophospholipids, like LPG, LPS, and LPI, oxylipins, and ceramide-1-phosphates were present at very low levels. Overall, lysoglycerophospholipids were the most abundant lipid category, making up over 88% of the plasma signaling lipidome, whereas the least abundant lipid category of endocannabinoids only accounted for 0.04% of the total lipid amount (Figure 5B). Considering the intersubject variability of human plasma, these concentrations are generally in good agreement with previously reported concentrations for plasma and NIST reference material. Comparing the herein reported concentrations of abundant lipid species to previous reports by Bowden *et al.*(23) and Medina *et al.*(22) shows high correlations of *R*^2^ > 0.96 and *R*^2^ > 0.84, respectively (Figure S9 and Table S16). But also low-abundant lipids like S1P 18:1;O2 are in good agreement with the literature, where values in the high nanomolar range have been reported.^23,27^

**Figure 6:**
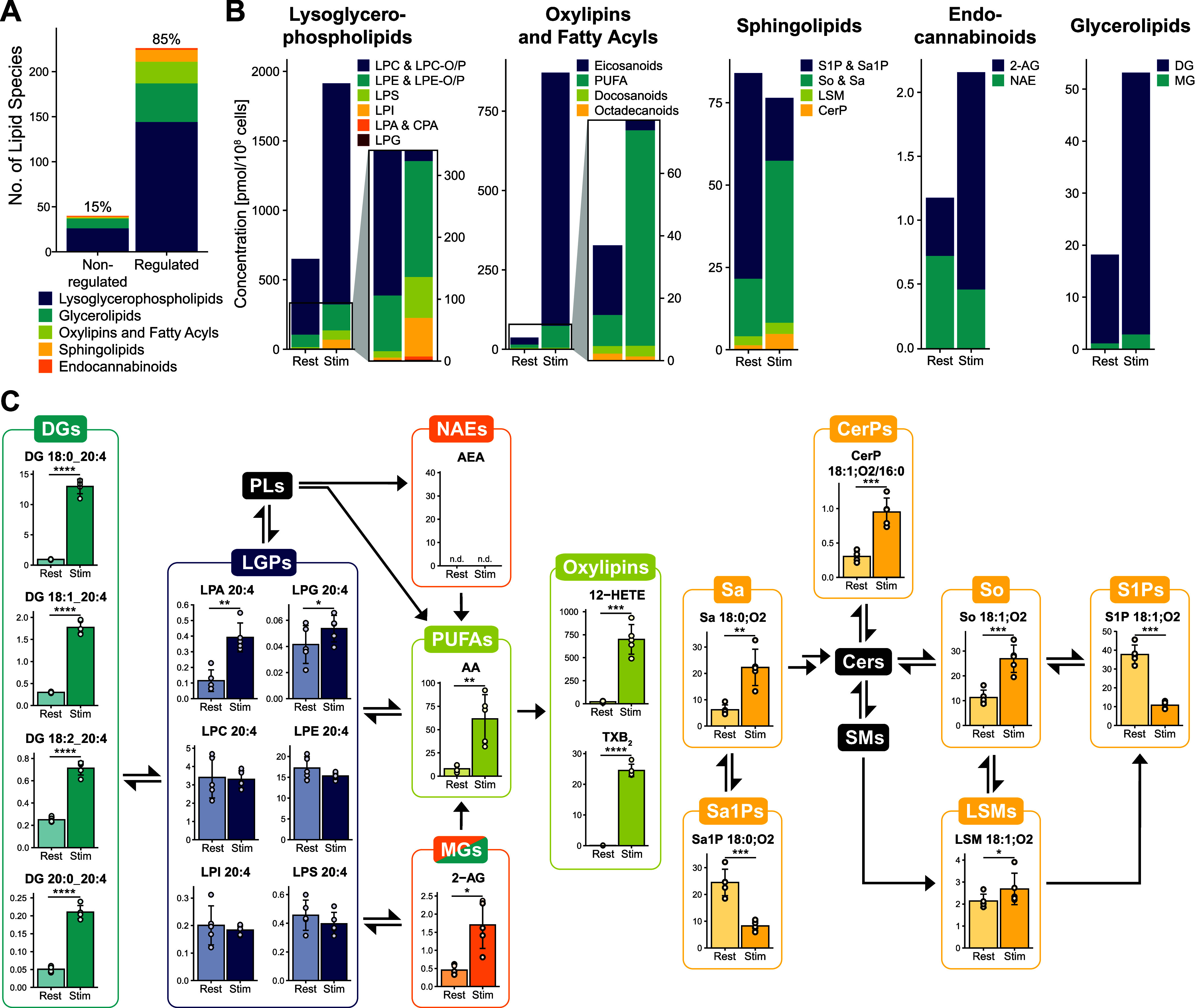
Analysis of the signaling lipidome in platelets reveals drastic regulation of cellular lipid signaling pathways during platelet activation (*n* = 5). Platelets were stimulated using 1 U/mL thrombin and 5 μg/mL collagen-related peptide for 5 min. (A) Bar graph depicting regulated and nonregulated lipids upon platelet stimulation. (B) Quantitative lipid changes of different lipid categories during platelet activation. (C) The established LC–MS/MS method enables the monitoring of distinct lipid signaling pathways for glycerophospholipid-derived (here on the example of AA-containing) lipids, as well as sphingoid-based signaling lipids. Concentrations are reported as pmol/10^8^ cells. n.d. = not detected.

Plasma is a challenging matrix to analyze because of its wide dynamic range and high abundance of apolar lipids like TGs and CEs. To demonstrate that our method is also suitable for investigating complex and highly dynamic processes along lipid signaling pathways, we studied the signaling lipidome during the activation of murine platelets. Although membrane lipidome remodeling and oxylipin signaling during platelet activation have already been described,^9,44^ there is no comprehensive report of signaling lipid regulation during this process. Using our LC–MS/MS workflow, we were able to illustrate the drastic changes in the platelet signaling lipidome upon stimulation (Figures 6, S10 and Table S17). A total of 267 lipids spanning almost 6 orders of magnitude were quantified, of which 85% were regulated upon stimulation (Figure 6A). Activated platelets showed a strong increase in lysoglycerophospholipids, glycerolipids, endocannabinoids, oxylipins, and PUFAs, whereas the overall concentration of signaling sphingolipids slightly decreased due to the significant reduction of S1P levels (Figure 6B). The high coverage of our established method furthermore enables the detailed investigation of distinct lipid signaling pathways for glycerophospholipid-derived and sphingoid-based signaling lipids on a molecular lipid species level. This is exemplified by arachidonic acid (AA)-containing lipid species in the platelet (Figure 6C). AA-containing lipids are of special interest as they serve as precursor molecules for generating important oxylipins during platelet activation.^9^ In line with the previously published results,^9,44−46^ platelet activation led to a significant cytosolic phospholipase A_2_-mediated^44^ (cPLA_2_) upregulation of LPA 20:4, AA, and AA-derived oxylipins. Furthermore, AA-containing DGs and 2-AG showed significantly increased concentrations likely mediated through the interplay of important lipid metabolic enzymes such as cPLA_2_ and phospholipase Cβ and γ.^9,45,47,48^ Sphingoid-based signaling lipids were also strongly regulated, with S1P and Sa1P being significantly downregulated in the platelets and upregulated in the releasate (Figure S10), indicating their release during platelet activation.^49^ CerPs, another important group of sphingoid-based signaling molecules,^6^ were also significantly induced upon activation. Notably, no CerP concentrations have ever been reported for platelets, making this the first publication to provide a detailed account of their regulation during platelet activation.

## Conclusions

Various challenges including isomeric and isobaric species, in-source fragmentation, and free phosphate group-containing analytes complicate a detailed investigation of the signaling lipidome. Here, we present a comprehensive LC–MS/MS method that overcomes these challenges by utilizing a bioinert column with optimized solvent and gradient composition. In conjunction with a simple yet robust sample preparation and targeted mass spectrometry, our analysis platform enables the monitoring of 388 lipid species from 17 different lipid classes with high sensitivity and repeatability and minimal analyte carryover even for free phosphate group-containing lipids like LPA, CerP, and S1P. To highlight the potential of our method for monitoring biological samples with complex lipid compositions and strong regulatory events, we investigated the signaling lipidome of plasma and platelets. Applying our method to plasma samples of 19 healthy subjects, 307 lipids spanning a dynamic range of 6 orders of magnitude were quantified. A similar wealth of lipid species was observed for the platelet signaling lipidome, revealing strong regulation of lipids along important lipid synthesis pathways during platelet activation. 85% of the 267 quantified lipids were regulated, with most signaling lipid categories (*e.g*. lysoglycerophospholipids, oxylipins, and glycerolipids) significantly increasing upon platelet stimulation. These two applications highlight the versatility of the herein presented method, making it a suitable choice for investigating signaling lipids in various matrix types. Monitoring 388 lipids in one 20 min run, our method enables the investigation of the signaling lipidome in unprecedented detail while still achieving similar sensitivity as other methods which are restricted to the selective analysis of only small subsets of signaling lipids.^8,15,25,27^

## Supporting Information Available

List of (internal) standards, instrument parameters, SRM transition list, lipid nomenclature, method characterization parameters, retention time stability data, lipidome comparison for NIST SRM 1950, and lipid concentrations in plasma and platelets (XLSX)
Sample generation and lipid extraction protocols, method characterization parameters, figures illustrating the chromatographic separation, ECN plots, calibration curves, figure illustrating the retention time stability, lipidome comparison plots, and figure illustrating the platelets releasate signaling lipidome (PDF)
