# Supplementary material for: A Targeted, Bioinert LC–MS/MS Method for Sensitive, Comprehensive Analysis of Signaling Lipids
Source: Anal Chem. 2024 May 25;96(23):9643–52. doi: 10.1021/acs.analchem.4c01388 (PMC11170558; doi:10.1021/acs.analchem.4c01388)
Supplement: Supplementary file 2 — ac4c01388_si_002.pdf [file ac4c01388_si_002.pdf]

Supporting Information

A targeted, bioinert LC-MS/MS method for sensitive, comprehensive analysis of signaling lipids

Stefanie Rubenzucker[1,2], Mailin-Christin Manke[3, 4], Rainer Lehmann[5], Alice Assinger[6], Oliver Borst[3, 4], Robert Ahrends[1]*

1 Department of Analytical Chemistry, University of Vienna, 1090 Vienna, Austria

2 Vienna Doctoral School in Chemistry, University of Vienna, 1090 Vienna, Austria

3 DFG Heisenberg Group Cardiovascular Thromboinflammation and Translational Thrombocardiology, University of Tübingen, 72076 Tübingen, Germany.

4 Department of Cardiology and Angiology, University of Tübingen, 72076 Tübingen, Germany.

5 Institute for Clinical Chemistry and Pathobiochemistry, Department for Diagnostic Laboratory Medicine, University Hospital Tübingen, 72076 Tübingen, Germany

6 Department of Vascular Biology and Thrombosis Research, Centre of Physiology and Pharmacology, Medical University of Vienna, 1090 Vienna, Austria

*Corresponding author: robert.ahrends@univie.ac.at

Table of Contents

Text S1 Generation of platelet and plasma samples. **S3**

Text S2 Lipid extraction protocols. **S4**

Text S3 Method characterization. **S5**

Figure S1 Influence of different column coatings on the separation of the critical pair PGE_2/PGD_2. .. **S6**

Figure S2 Chromatographic separation of (A) lysoglycerophospholipids and (B) the critical oxylipin pair PGE_2/PGD_2 in plasma. **S7**

Figure S3 Equivalent carbon number (ECN) model plots. **S8**

Figure S4 Calibration curves in neat solvent.. **S10**

Figure S5 Calibration curves in plasma. **S12**

Figure S6 Calibration curves in platelet pellet. **S14**

Figure S7 Calibration curves in platelet supernatant. **S16**

Figure S8 Retention time stability of the LC-MS/MS method over multiple weeks and different matrices for selected analytes (n=188)... **S18**

Figure S9 Comparison of plasma lipid concentrations to lipid concentrations measured in NIST SRM 1950 plasma by Bowden *et al.* and Medina *et al.*.. **S19**

Figure S10 Analysis of the signaling lipidome in platelets during platelet activation (n=5).............. **S20**

Text S1 Generation of platelet and plasma samples.

Platelets

Mice were anesthetized and blood was drawn from the retrobulbar plexus into citrate anticoagulated tubes. Platelet-rich plasma (PRP) was obtained by centrifuging at 264 g for 5 min followed by 52 g for 6 min. Afterwards, PRP was centrifuged at 640 g for 5 min to pellet the platelets. After a washing step, the obtained platelet pellet was resuspended in modified Tyrode-HEPES buffer, pH 7.4 (137 mM NaCl, 12 mM $NaHCO_3$, 2 mM KCl, 5.5 mM glucose, 0.3 mM NaH_2PO_4, 5 mM HEPES, 1 mM $CaCl_2$). The cell count was determined using a Sysmex automatic hematology analyzer (Norderstedt, Germany), with one sample equaling 10^8 platelets. The freshly isolated platelets were then either left in a resting state or stimulated with 5 µg/mL CRP and 1 U/mL thrombin for 5 min. The pellet and releasate were separated by centrifuging for 5 min at 640 g and RT and subsequently separately shock frozen in liquid nitrogen and stored at -80 °C until further use. All animal experiments were performed according to the Directive 2010/63/EU of the European Parliament on the protection of animals used for scientific purposes and approved by local authorities (Regierungspräsidium Tübingen) following the ARRIVE guidelines.

Plasma

Blood was drawn into 3.8% sodium citrate tubes and centrifuged at 1,000 g and 4 °C for 10 min. The plasma supernatant was transferred to a new vial and subjected to a second round of centrifugation at 10,000 g and 4 °C for 10 min to guarantee the removal of all cellular components. The supernatant was then stored in aliquots at −80 °C until analysis. The study has been approved by the ethics committee of the Medical University of Vienna (EKNr:1548/2020) in conformity with all European, national and institutional guidelines, including the Declaration of Helsinki and the Conventions of the Council of Europe on Human Rights and Biomedicine. Written informed consent was obtained before sampling.

Text S2 Lipid extraction protocols.

BuEt two-phase extraction

5 µL IS mix and 350 µL 200 mM citric acid + 100 mM K_2HPO_4 (pH 4.5) were added to 100 µL thawed plasma. 1 mL BuOH/EtOAc 1/1 (v/v) was added and the sample was thoroughly mixed using a vortex mixer and subsequently ultrasonicated on ice for 10 s. After vigorous mixing for another 2 min, the sample was centrifuged at 4 °C and 21,000 g for 10 min. 900 µL of the upper organic phase were collected and the lower phase was re-extracted using 400 µL water-saturated BuOH and 400 µL EtOAc. The combined organic phases were dried under a nitrogen stream.

BuMe one-phase extraction

5 µL IS mix and 1 mL BuOH/MeOH 3/1 (v/v) were added to 100 µL thawed plasma. The sample was thoroughly mixed using a vortex mixer and subsequently ultrasonicated on ice for 10 s. After mixing for another 10 s, the sample was incubated at 4 °C and 950 rpm for 30 min. The protein fraction was precipitated by centrifuging at 4 °C and 21,000 g for 15 min. The organic phase was recovered and dried under a nitrogen stream.

MMC one-phase extraction

5 µL IS mix and 1 mL MeOH/MTBE/$CHCl_3$ 1.33/1/1 (v/v/v) were added to 100 µL thawed plasma. The sample was thoroughly mixed using a vortex mixer and subsequently ultrasonicated on ice for 10 s. After mixing for another 10 s, the sample was incubated at 4 °C and 950 rpm for 30 min. The protein fraction was precipitated by centrifuging at 4 °C and 21,000 g for 15 min. The organic phase was recovered and dried under a nitrogen stream.

Text S3 Method characterization.

Sensitivity, linearity, and matrix effect

Sensitivity, linearity, and matrix effect were determined by spiking a mix of 27 non-endogenous internal standards at different concentration levels (Table S8). These standard calibration curves were prepared in triplicates in solvent and matrix-matched in plasma and platelet matrix. The matrix effect was calculated as the percentage of the ratio between the slope of the matrix-matched and the slope of the solvent calibration curve. The limit of detection (LOD) was determined as the lowest calibration standard yielding a signal-to-noise ratio (S/N) ≥ 3 and the lower limit of quantification (LLOQ) was defined as the calibration standard yielding a S/N ≥ 5. S/N ratios were calculated using Analyst® 1.7.2 (AB Sciex, Concord, Canada). Calibration curves were calculated using weighted linear least squares regression (weighting $1/x^2$) in OriginPro Version 2020 (OriginLab Corporation, Northampton, USA).

Precision

Inter- and intraday precision was evaluated in triplicate for the 50 nM standard and is expressed as the coefficient of variation (CV) of three replicate measurements. Interday precision corresponds to the measurement of three independent samples over one week, intraday precision to the measurement of three independent samples on the same day.

Carryover

The carryover effect was determined by injecting solvent blanks directly after the highest concentrated calibrant. The carryover is expressed as the percentage of the ratio between the standard peak area in the blank and the standard peak area in the highest calibrant.

Retention time stability

Instrument repeatability was assessed by monitoring the retention time reproducibility of selected endogenous analytes in different matrices throughout multiple batches over several weeks.

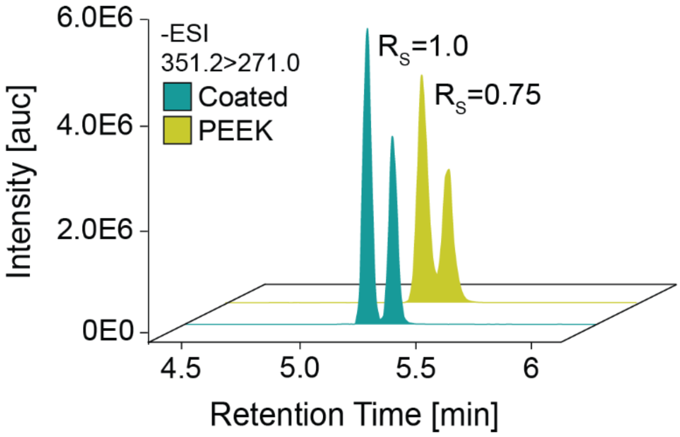

Figure S1 Influence of different column coatings on the separation of the critical pair PGE_2/PGD_2.

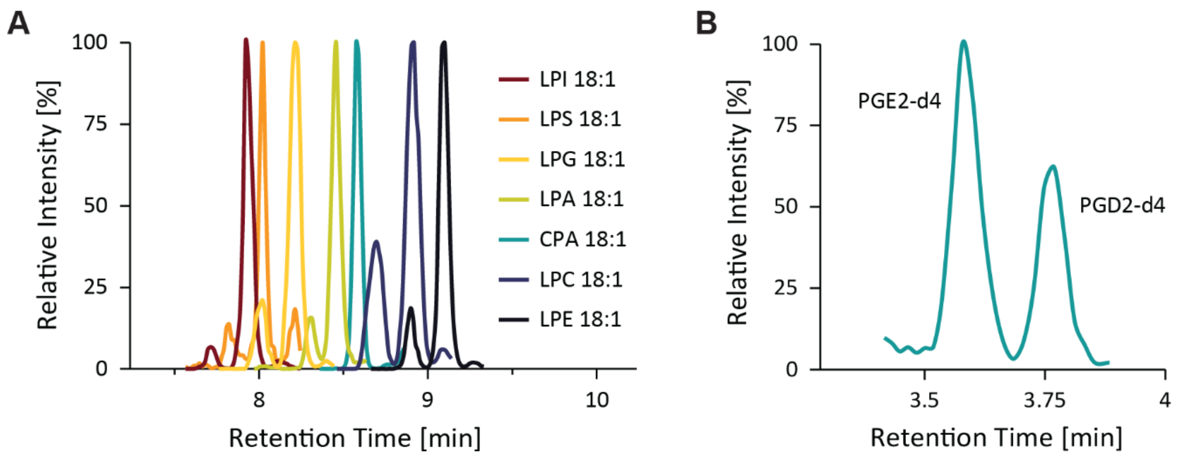

Figure S2 Chromatographic separation of (A) lysoglycerophospholipids and (B) the critical oxylipin pair PGE_2/PGD_2 in plasma. Separation of CPA and LPS from LPA is crucial due to possible in-source fragmentation. Furthermore, PGE_2/PGD_2 also need to be chromatographically separated, as they do not possess unique MS/MS fragments.

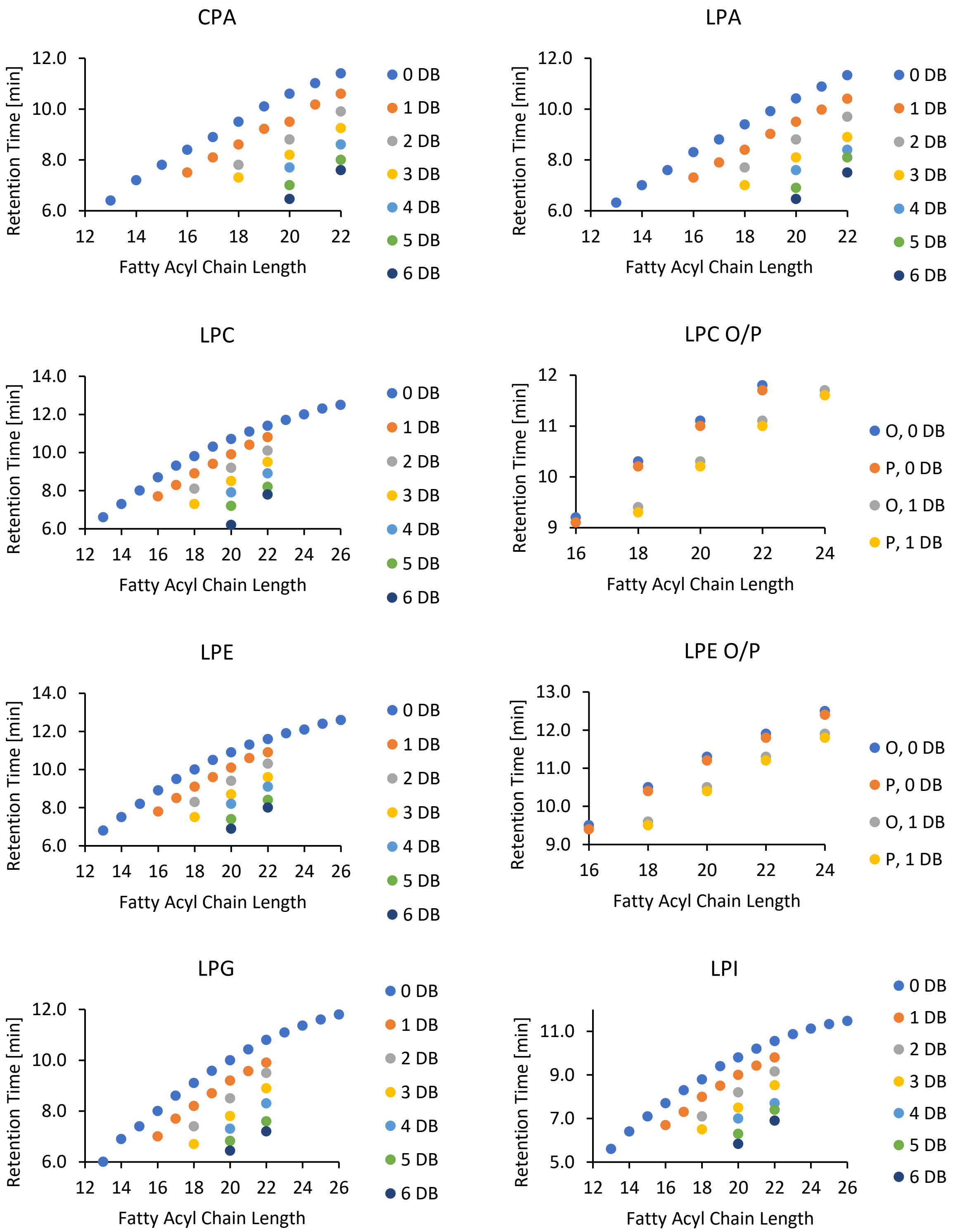

Figure S3 Equivalent carbon number (ECN) model plots.

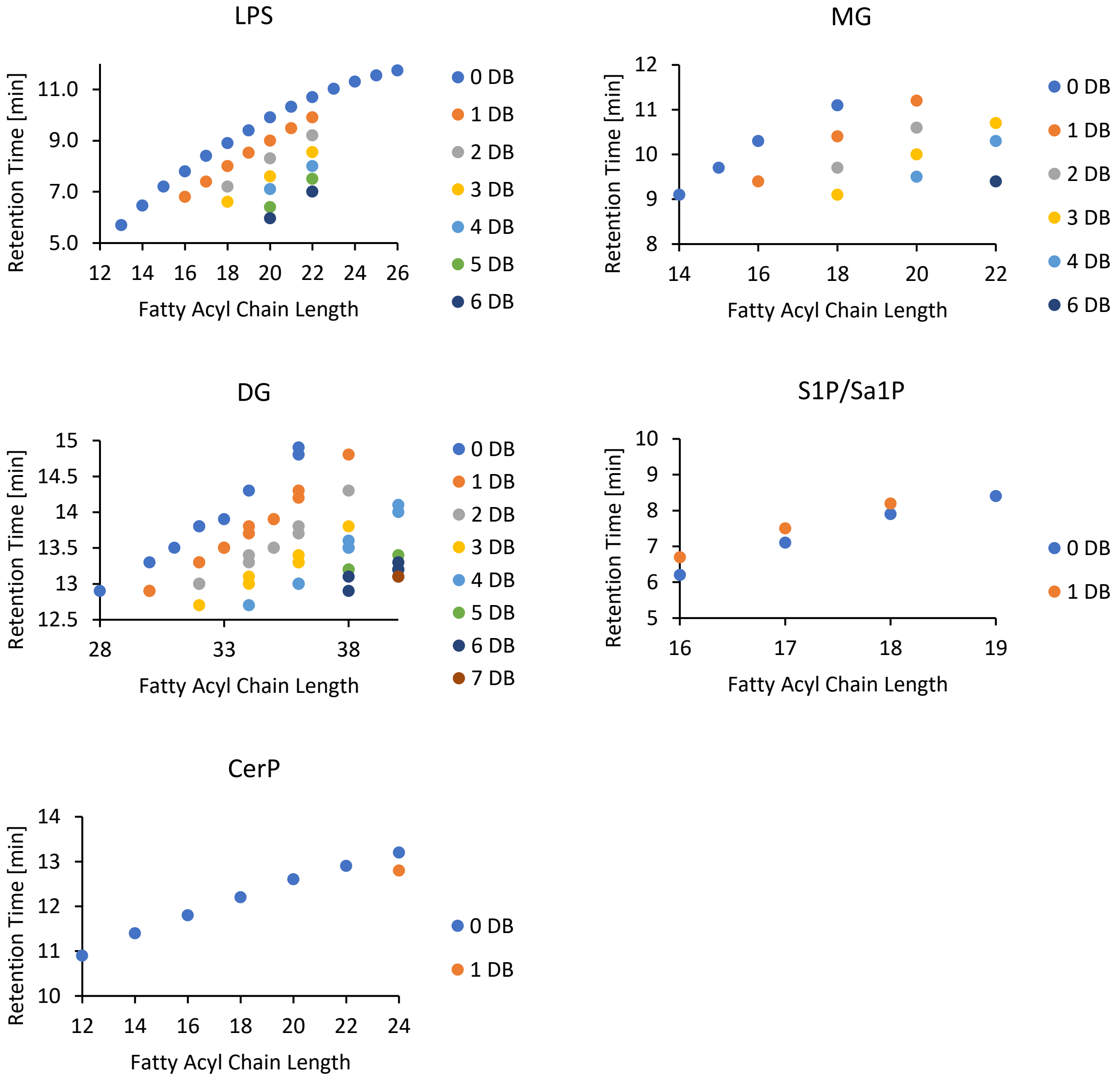

Figure S3 Continued.

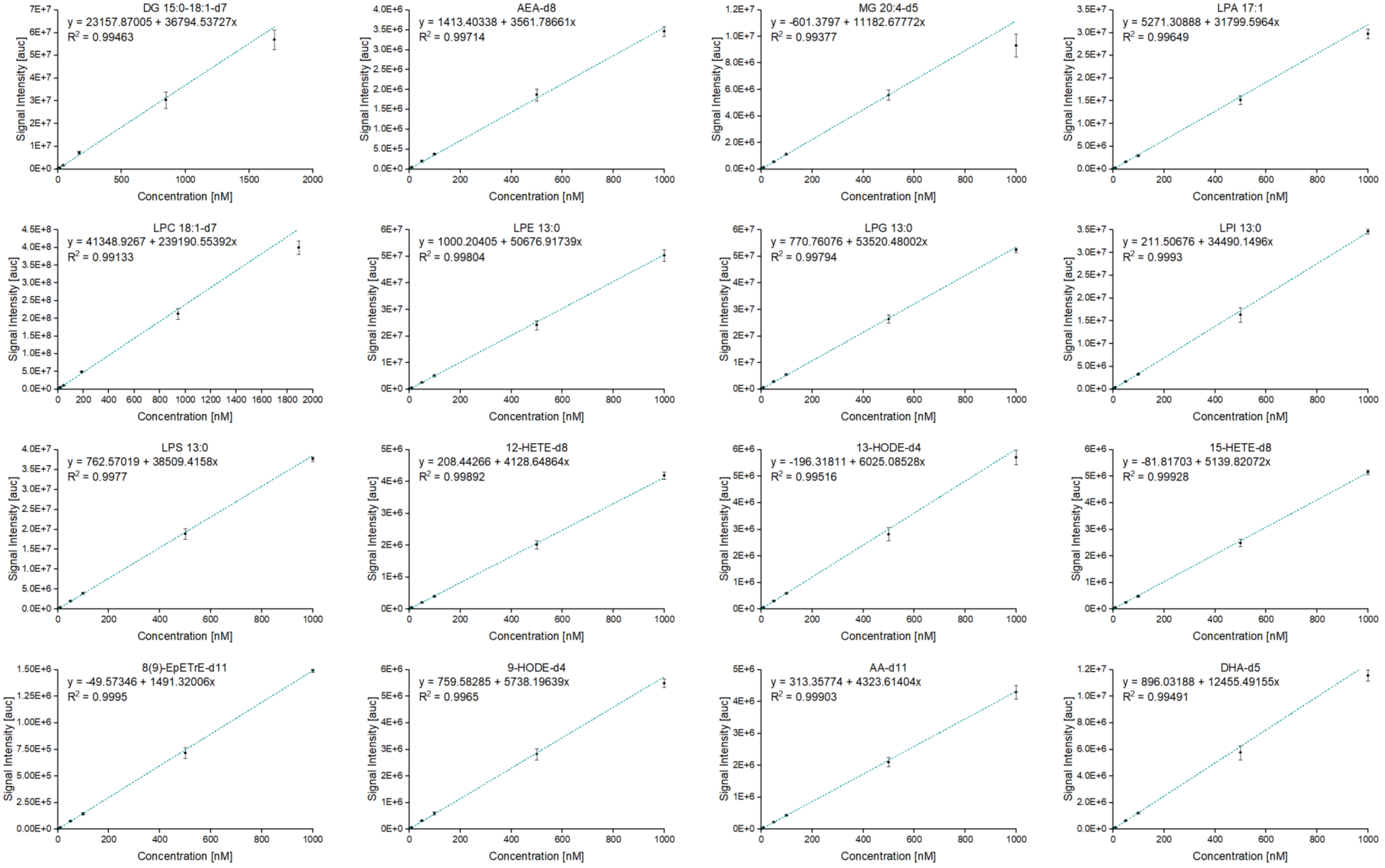

Figure S4 Calibration curves in neat solvent.

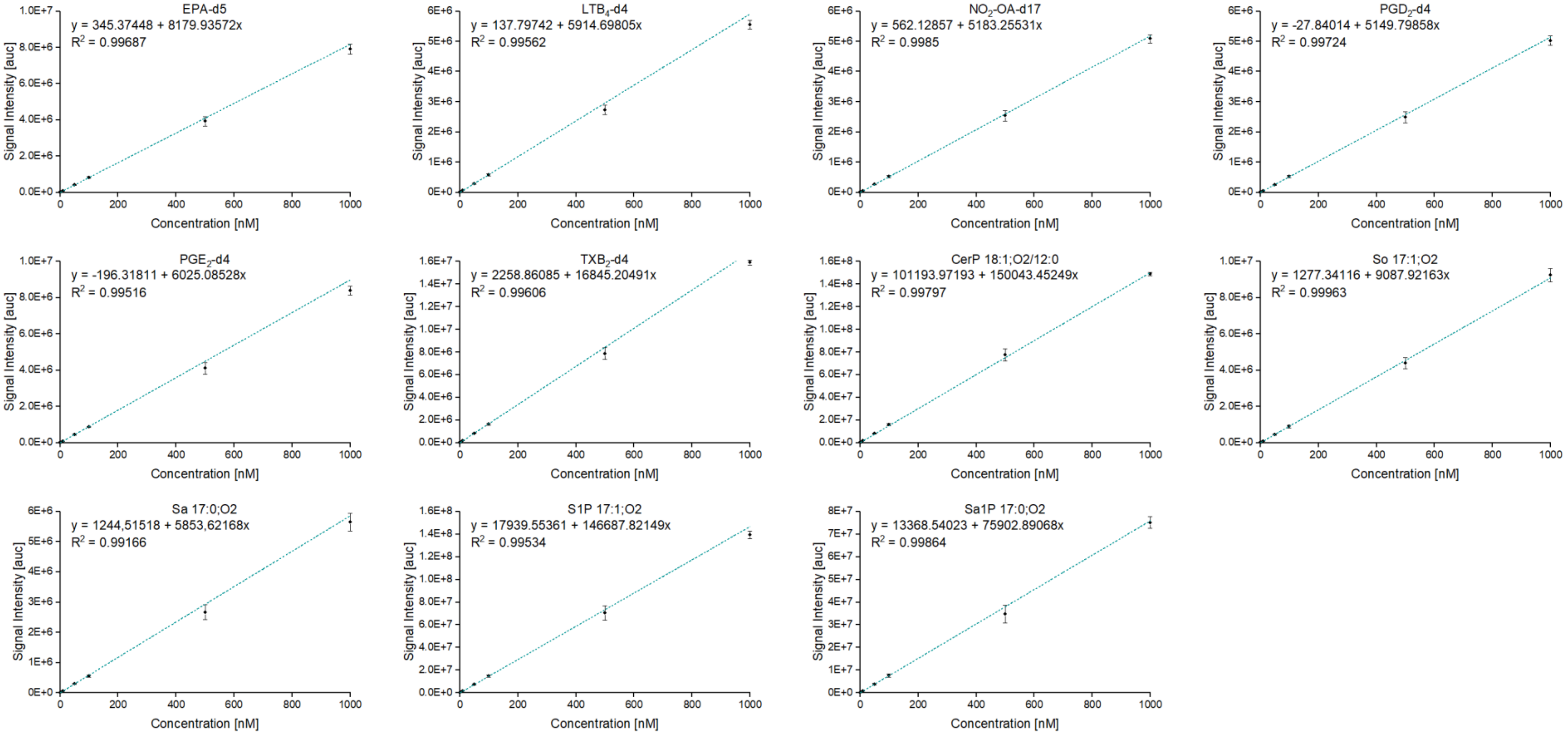

Figure S4 Continued.

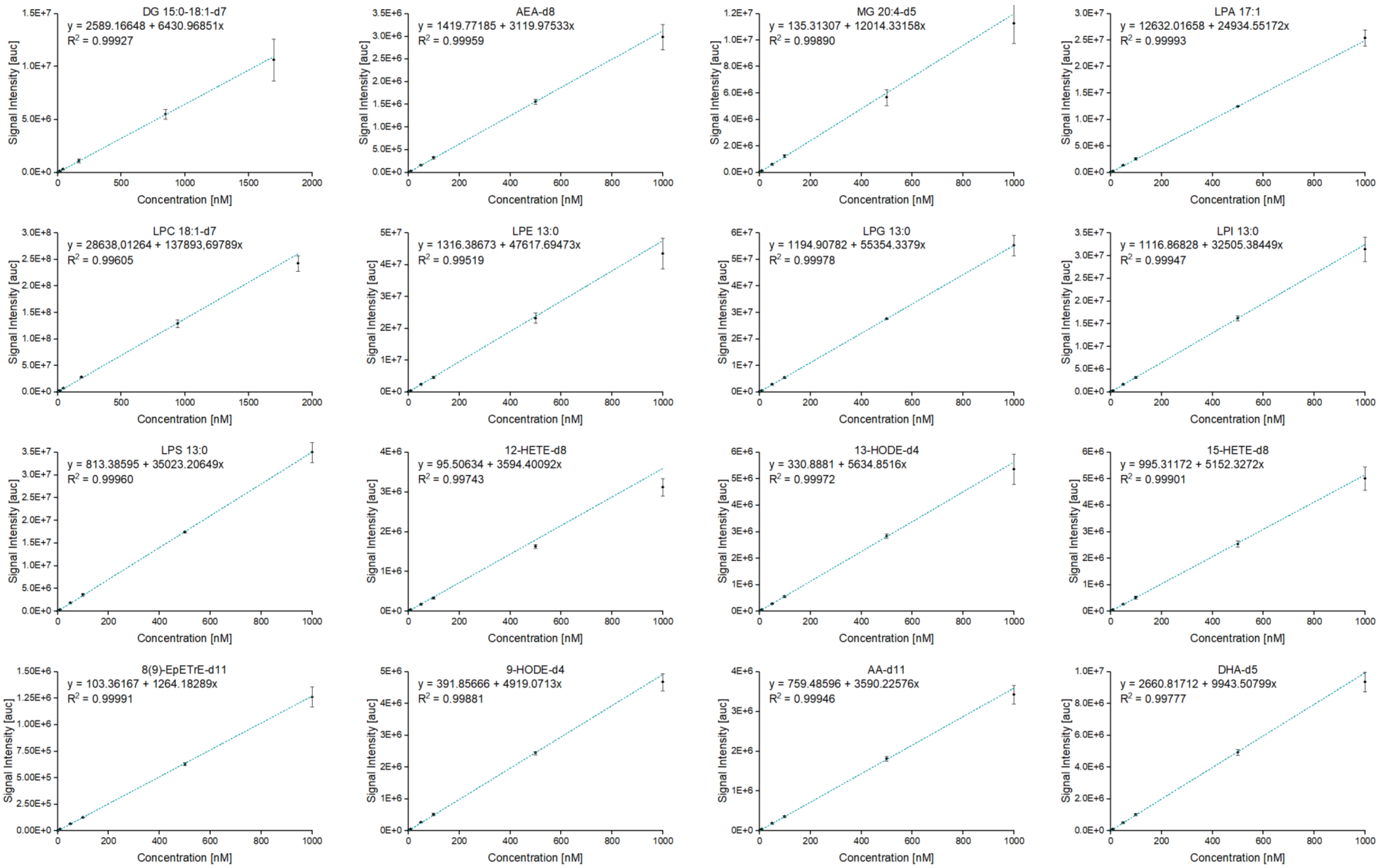

Figure S5 Calibration curves in plasma.

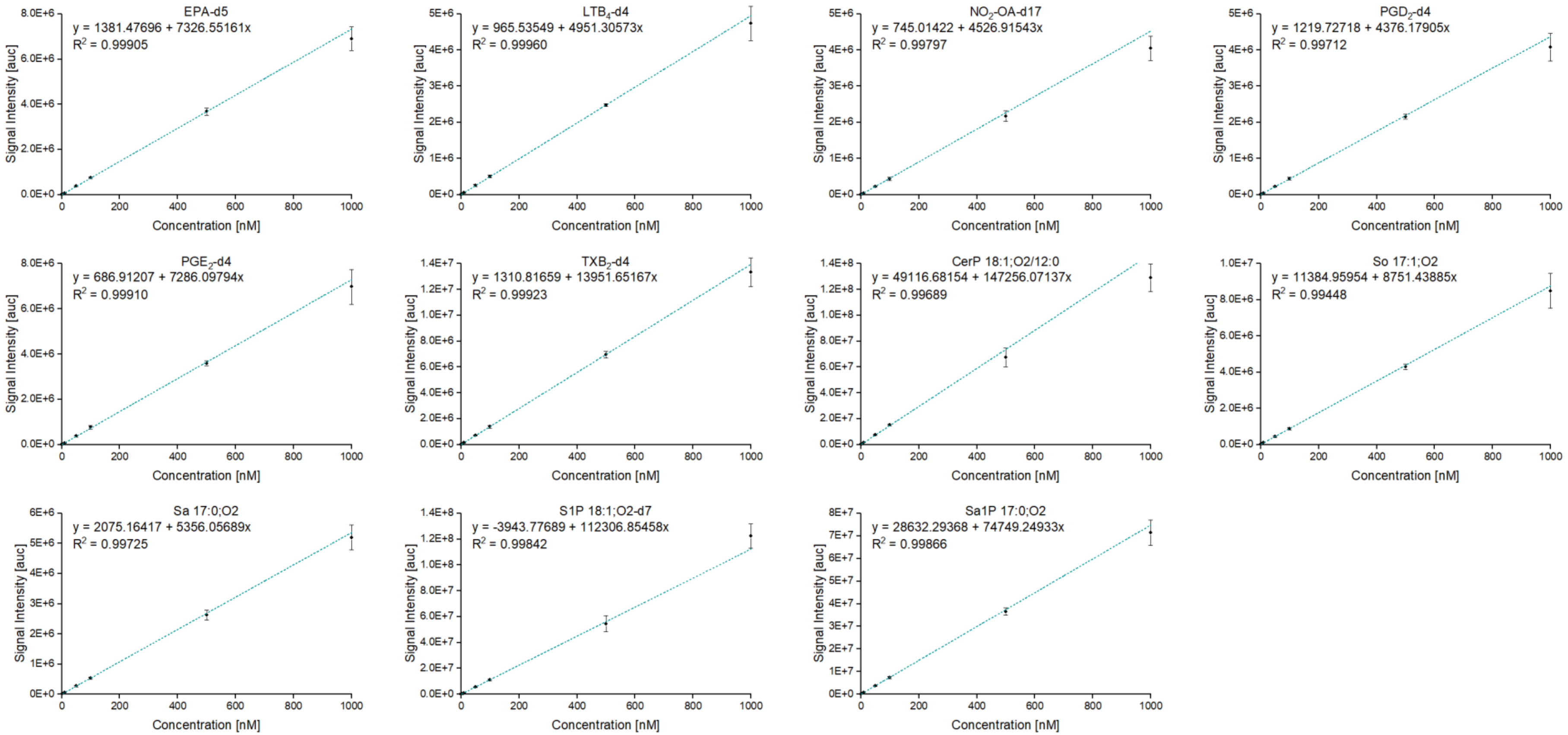

Figure S5 Continued.

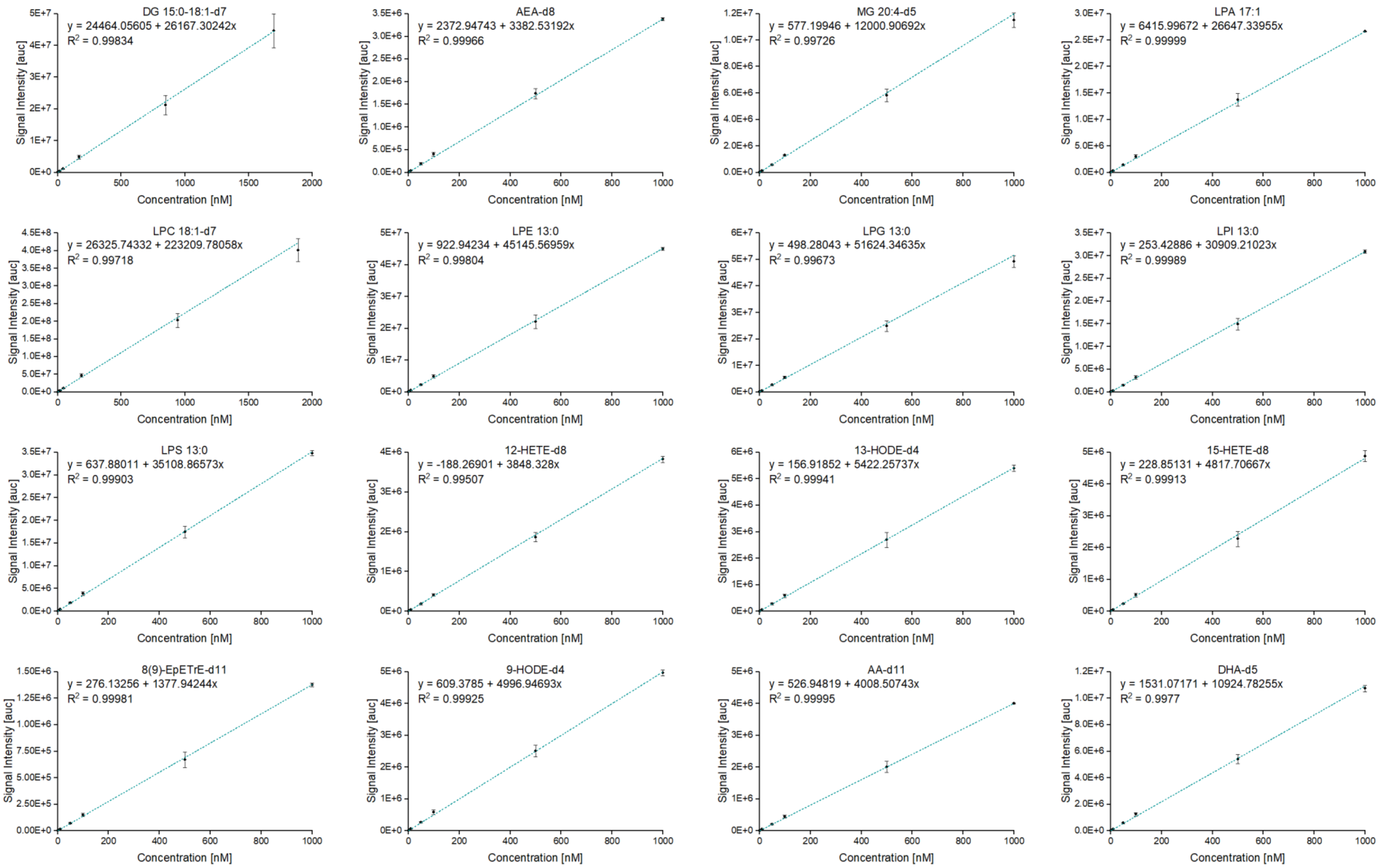

Figure S6 Calibration curves in platelet pellet.

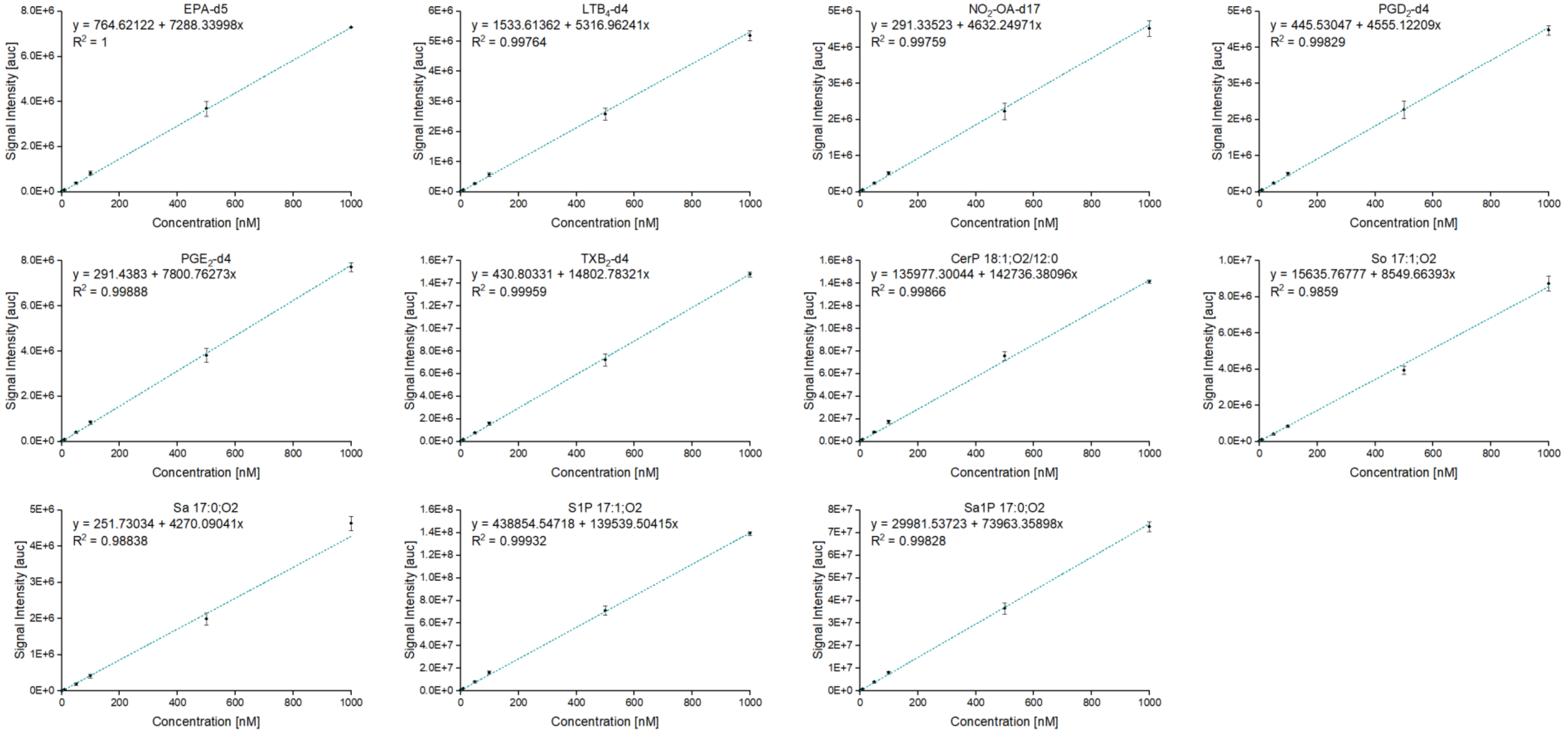

Figure S6 Continued.

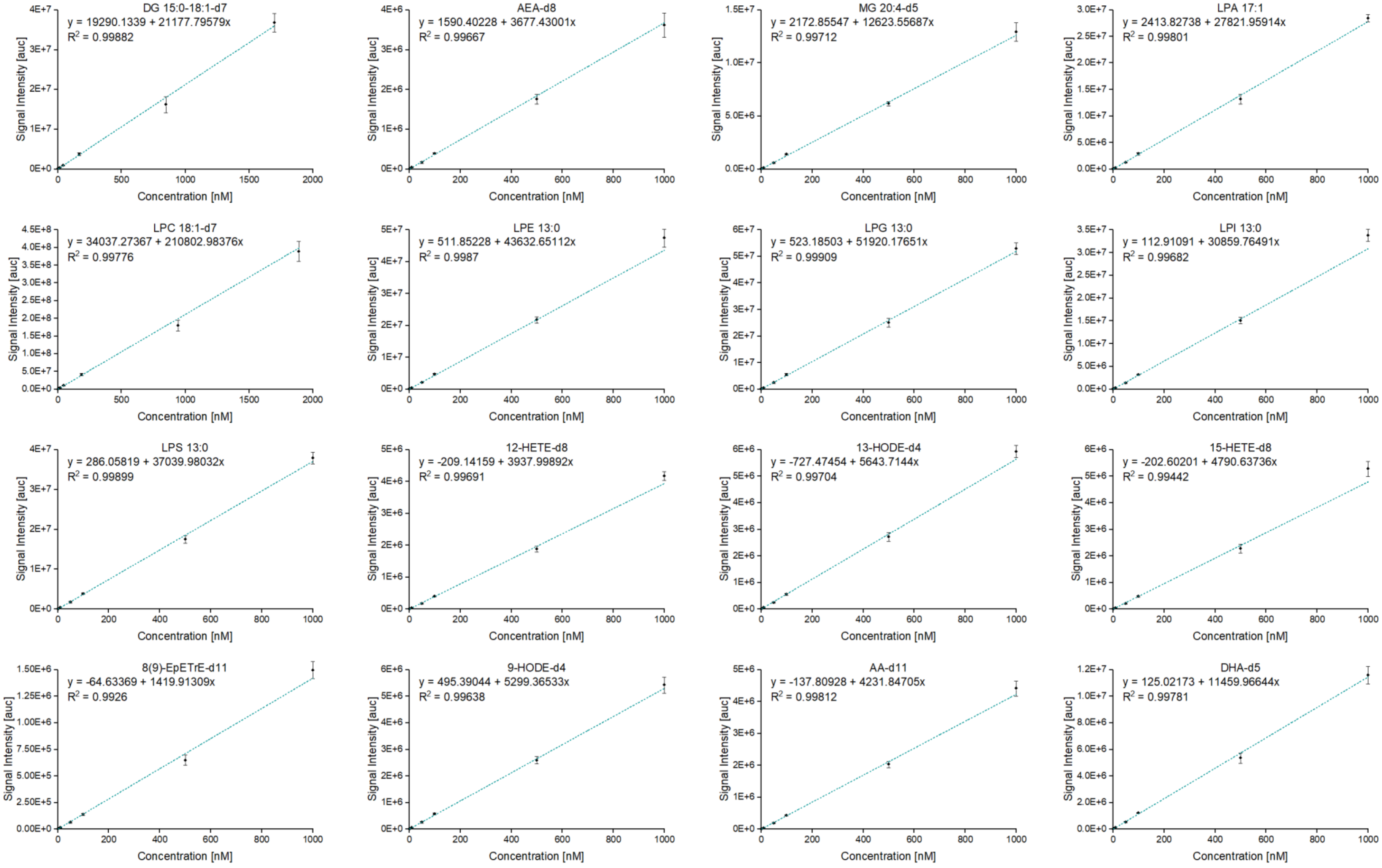

Figure S7 Calibration curves in platelet supernatant.

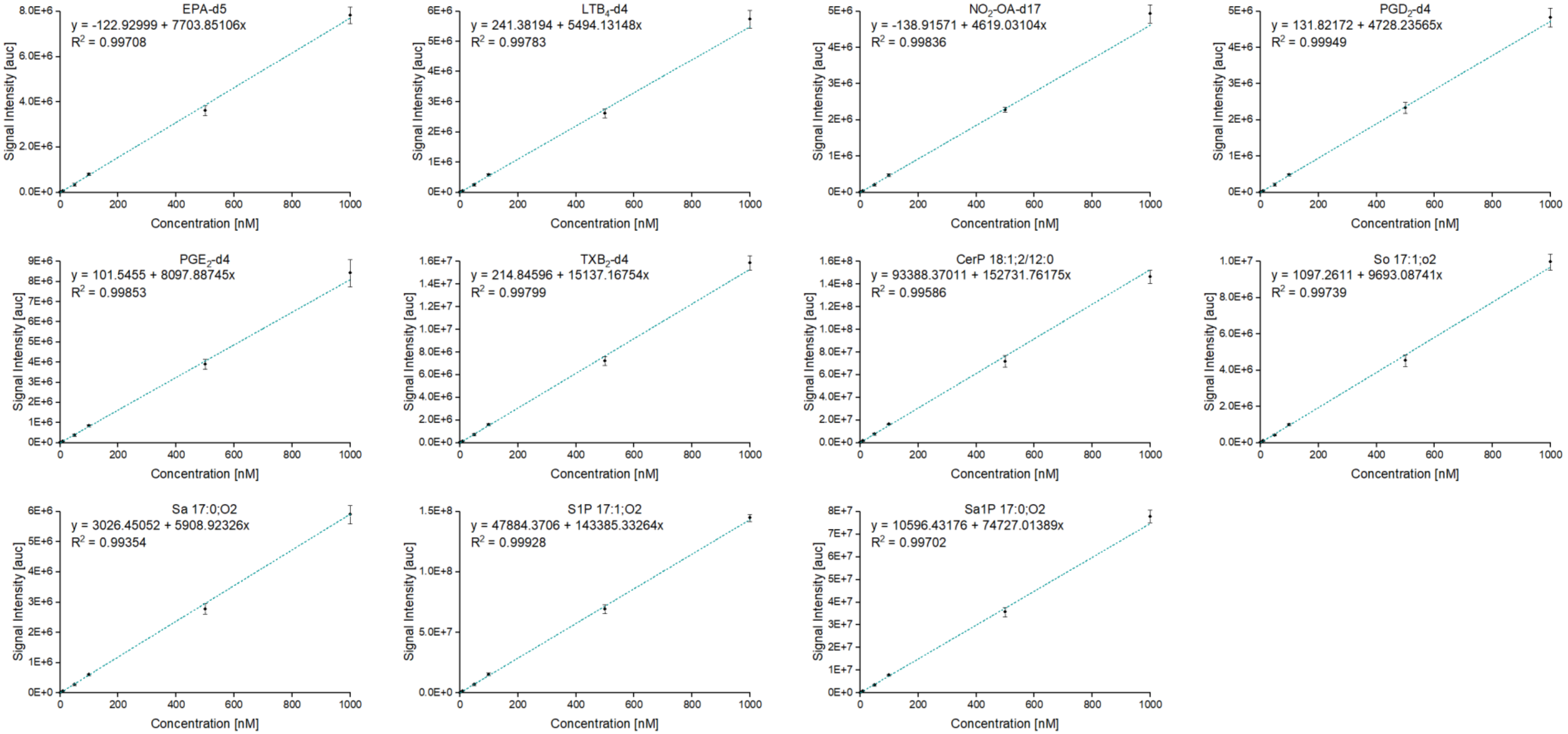

Figure S7 Continued.

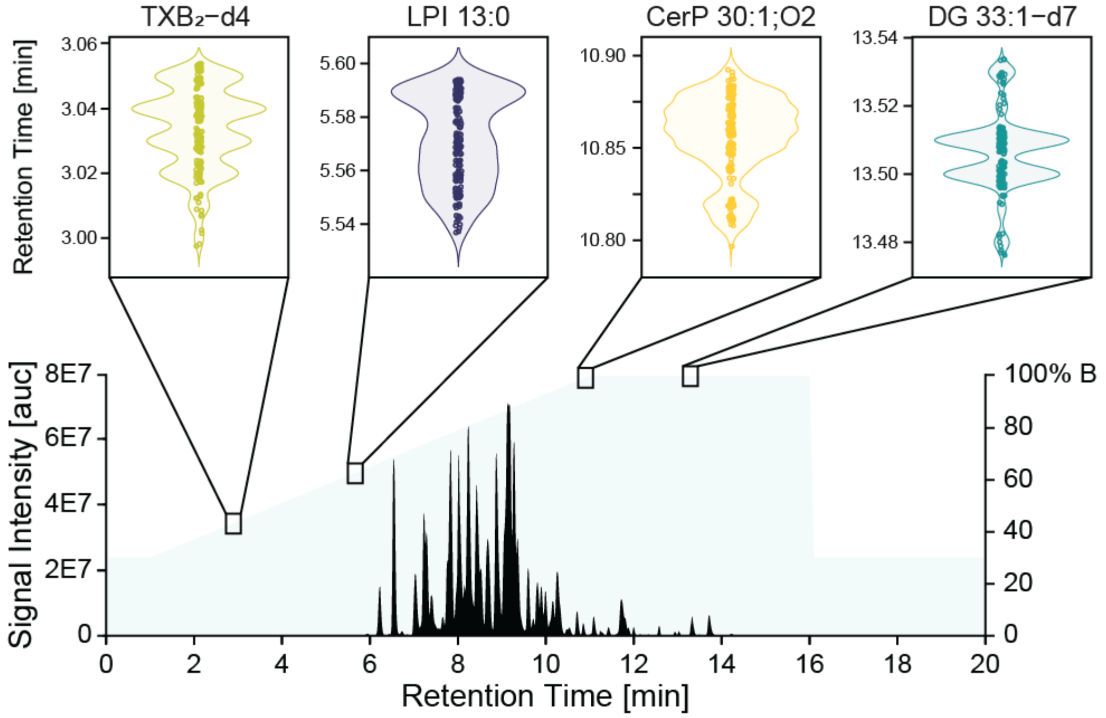

Figure S8 Retention time stability of the LC-MS/MS method over multiple weeks and different matrices for selected analytes (n=188).

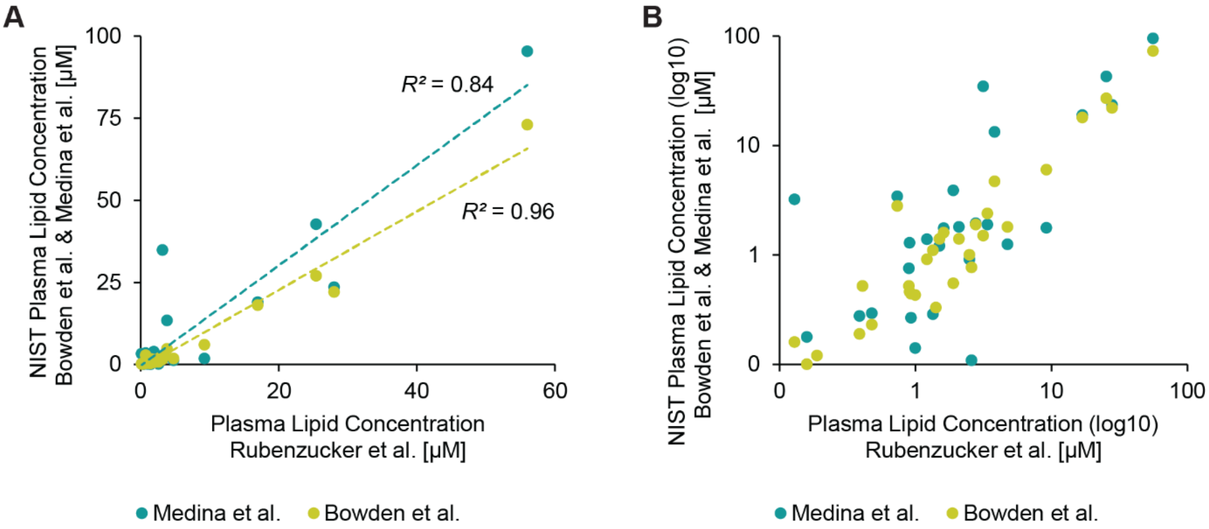

Figure S9 Comparison of plasma lipid concentrations to lipid concentrations measured in NIST SRM 1950 plasma by Bowden *et al.* (https://doi.org/10.1194/jlr.M079012) and Medina *et al.* (https://doi.org/10.1021/acs.analchem.2c02598). All species shared between the three publications have been used for comparison (n=30). For better visualization, concentrations were plotted (A) on a linear scale and (B) on a log10 scale.

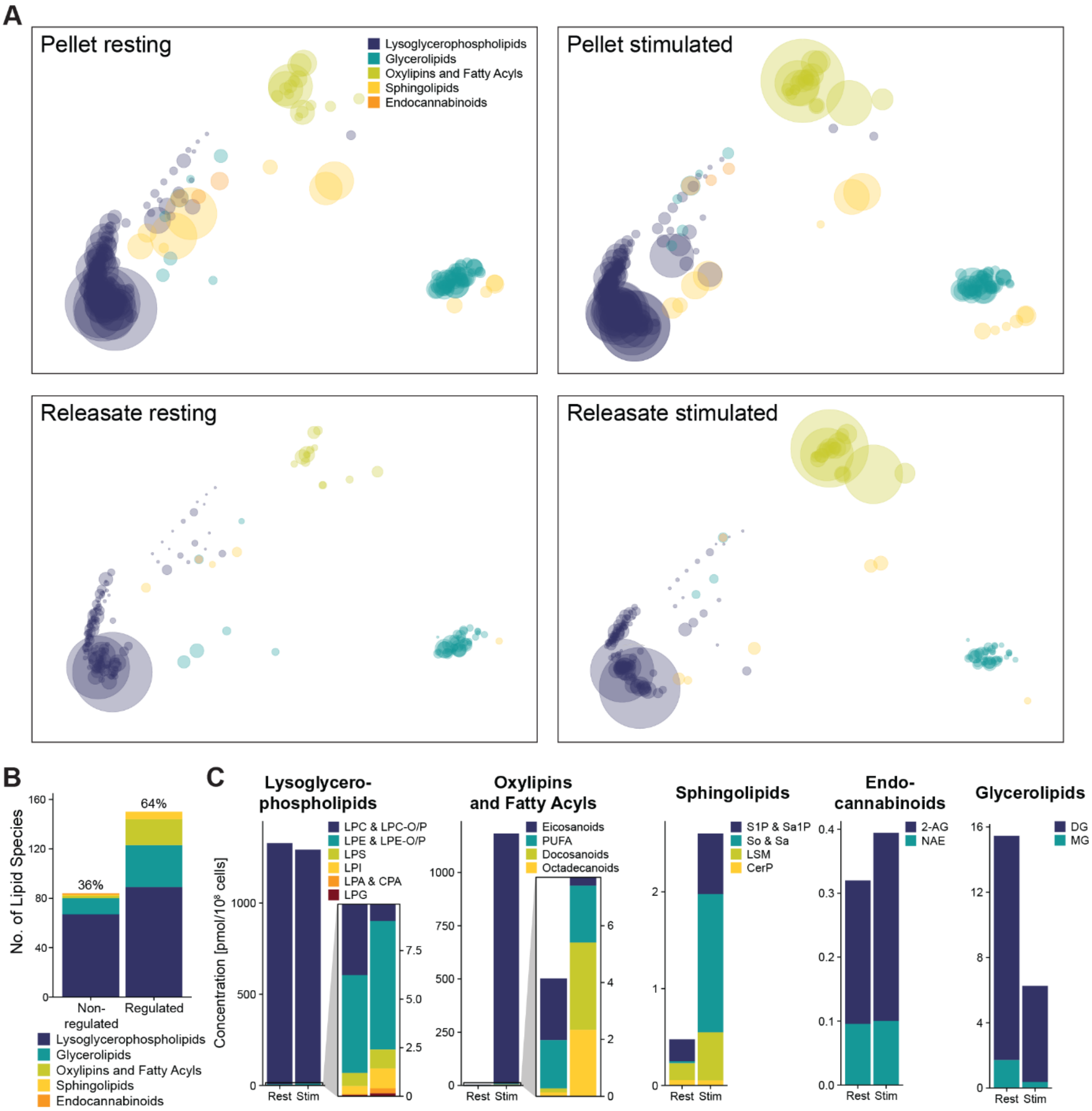

Figure S10 Analysis of the signaling lipidome in platelets during platelet activation (n=5). Platelets were stimulated using 1 U/mL thrombin and 5 µg/mL CRP for 5 min. (A) Lipid spaces illustrating the regulation of the signaling lipidome in platelet and their releasates during platelet activation. (B) Bar graph depicting regulated and non-regulated lipids in the platelet releasate upon stimulation. (B) Quantitative lipid changes of different lipid categories in the platelet releasate during platelet activation
